# Lung Cancer Patients Display Increased Expression of Bovine Meat and Milk Factor (BMMF) Proteins in Peritumor Alveolar Macrophages

**DOI:** 10.1002/ijc.70574

**Published:** 2026-06-01

**Authors:** Nives Cecere, Kristina Alikhanyan, Claudia Ernst, Lisa Häfele, Lars Feuerbach, Imke Grewe, Sumen Siqin, Karin Gunst, Gunjan Shukla, Veronika Frehtman, Claudia Tessmer, Florian Eichhorn, Michael Allgäuer, Alexander Brobeil, Ilse Hofmann, Barbara Leuchs, Marc A. Schneider, Timo Bund

**Affiliations:** ^1^ Division of Episomal‐Persistent DNA in Cancer‐ and Chronic Diseases German Cancer Research Center (DKFZ) Heidelberg Germany; ^2^ Division of Applied Bioinformatics German Cancer Research Center (DKFZ) Heidelberg Germany; ^3^ Biopharmaceutical Production and Development Unit German Cancer Research Center (DKFZ) Heidelberg Germany; ^4^ Core Facility Antibodies German Cancer Research Center (DKFZ) Heidelberg Germany; ^5^ Department of Thoracic Surgery Thoraxklinik at Heidelberg University Hospital Heidelberg Germany; ^6^ Translational Lung Research Center Heidelberg (TLRC), Member of the German Center for Lung Research (DZL) Heidelberg Germany; ^7^ Department of Pathology Heidelberg University Hospital Heidelberg Germany; ^8^ Tissue Bank of the National Center for Tumor Diseases (NCT) Heidelberg Germany; ^9^ Translational Research Unit Thoraxklinik at Heidelberg University Hospital Heidelberg Germany

**Keywords:** bovine meat and milk factors, chronic inflammation, dietary cancer risk factors, indirect carcinogenesis, lung cancer

## Abstract

Besides smoking and exposure to specific environmental risk factors, also diet and in particular red meat consumption/exposure are described as risk factors contributing to lung cancer development. Incidence of non‐smoking associated lung cancer is rising rapidly, globally. Global epidemiology supports an association between lung cancer incidence and consumption of bovine products. The search for meat‐/milk‐associated cancer risk factors led to the isolation of plasmid DNA termed Bovine Meat and Milk Factors (BMMFs). Due to detection of increased expression levels of a conserved BMMF‐encoded protein (Rep) in tissues of colorectal cancer patients, BMMFs have been proposed as causal risk factors for cancer acting via inflammation‐driven indirect carcinogenesis. Yet, their presence and possible contribution to lung cancer remained elusive. In this study, Rep expression was quantified in paired tumor/peritumor tissues from lung cancer patients (*n* = 35) as well as cancer‐free individuals (*n* = 19, tissues adjacent to benign hamartoma) by immunohistochemistry, co‐immunofluorescence microscopy, immunodetection/mass spectroscopy and genomics/transcriptomics screening. In all patients tested, Rep expression was immunohistochemically observed in CD68^+^ CD163^+^ alveolar lung macrophages, which were present at higher numbers in peritumor tissues of cancer patients compared to controls. Expression was inversely correlated with smoking intensity, supporting BMMFs' function as a new, smoking‐independent biomarker and possible driver for lung cancer. BMMF^+^ macrophages revealed a cancer‐promoting M2‐like phenotype upon scRNA‐seq, suggesting BMMF as target for both prevention and therapy. BMMFs might contribute to lung cancer by inducing pro‐tumorigenic macrophage populations and serve as marker for (early) cancer detection and prevention.

## What's New?

Plasmid‐like DNA sequences frequently found in bovine milk and serum have been proposed as infectious agents promoting indirect carcinogenesis. This multi‐omics study detected immune cells positive for these bovine meat and milk factors (BMMFs) in patients with lung cancer, with BMMF expression inversely correlated with smoking intensity. Positive macrophages showed a M2‐like phenotype, suggesting BMMF as a target for both prevention and therapy. BMMFs might contribute to lung cancer by inducing pro‐tumorigenic macrophage populations and serve as a marker for cancer detection and prevention. The data might help explain the global increase of lung cancer cases among young, non‐smoking individuals.

## Graphical abstract

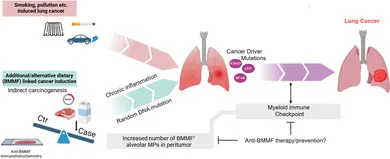

**Abbreviations**
8OHdG: 8‐hydroxy‐2′‐deoxyguanosine
AA: amino acids
AB: antibody
BMMF: Bovine Meat and Milk Factors
bp: base pair
BSA: bovine serum albumin
CD163: cluster of differentiation 163
CD45: leukocyte common antigen
CD68: cluster of differentiation 68
COPD: Chronic Obstructive Pulmonary Disease
CRC: colorectal cancer
CT: cycle threshold
DAB: Diaminobenzidine
DapB: Dihydrodipicolinate Reductase gene of *Bacillus subtilis*

D‐ViSioN: detection of integrated viral sequences by singletons
ELISA: Enzyme‐linked immunosorbent assay
FFPE: Formalin‐ Fixed Paraffin Embedded
GAPDH: Glyceraldehyde‐3‐phosphate dehydrogenase
HRP: horseradish peroxidase
IF: Immunofluorescence
IHC: immunohistochemistry
IP: immunoprecipitation
ISH: in situ hybridization
LUAD: lung adenocarcinoma
LUSC: lung squamous cell carcinoma
MP/MPs: macrophage(s)
MSBI: multiple sclerosis brain isolate
NSCLC: non‐small cell lung cancer
PBS: Phosphate‐buffered saline
PBST: PBS with Tween‐20
PCAWG: pan‐cancer analysis of whole genomes
PPIB: Peptidylprolyl Isomerase B
PY: pack‐years
Rep: replication‐associated protein
RIPA buffer: Radioimmunoprecipitation assay buffer
RNS: reactive nitrogen species
ROS: reactive oxygen species
RT‐qPCR: reverse transcription quantitative PCR
SCLC: small cell lung cancer
scRNA‐seq: single‐cell RNA sequencing
SDS‐PAGE: sodium dodecyl sulfate polyacrylamide gel electrophoresis
TAM: tumor‐associated macrophages
WB: western blot
WGS: whole genome sequencing

## 1 Introduction

Although up to 90% of lung cancer cases are linked to smoking, exposure to other environmental and dietary factors and chronic inflammation also contribute to lung cancer development [1]. Increased cancer incidence was found for butchers and meat workers [2, 3], and decreased incidence was reported in the context of lactose intolerance or usage of non‐steroidal anti‐inflammatory drugs [4, 5, 6]. Global epidemiology as well as the reduced incidence with lactose intolerance point toward an association between lung cancer and consumption of bovine products, which prompted the search for infectious agents in those products [4, 7, 8, 9].

Bovine Meat and Milk factors (BMMFs) are plasmid‐like DNA sequences frequently isolated from bovine milk and serum as well as from the peritumor of colorectal cancer (CRC) patients amongst others [10, 11, 12, 13, 14, 15, 16, 17, 18, 19, 20]. More than 180 BMMFs are known and classified in 4 different groups (“BMMF1‐4”) of Sphinx/BMMF‐like elements [8, 15]. In human cells, BMMFs replicate, transcribe and translate BMMF proteins [21]. BMMFs have been proposed as infectious agents and risk factors involved in the development of different types of cancer, including lung cancer, by inducing chronic inflammation and DNA mutation in exposed tissues via indirect carcinogenesis [4, 9, 22, 23]. Immunohistochemistry (IHC) with anti‐BMMF Rep antibodies (ABs) supported this model experimentally by revealing increased expression levels of a conserved, BMMF‐encoded protein (Rep) in the peritumor of CRC patients compared to healthy controls, thus indicating a role of BMMFs as biomarker for cancer [9, 22, 23]. Rep expression was associated with increased numbers of peritumor M2‐like CD68^+^/CD163^+^ macrophages (MPs), was spread widely over the colonic mucosa, and was already raised in pre‐cancerous low‐ and high‐grade dysplasia preceding CRC [23, 24].

Tumor‐associated macrophages (TAMs) in general and M2‐like TAMs in particular represent a major component of the tumor microenvironment, supporting progression of several types of tumors [25, 26]. This includes also lung cancer, where TAMs have been shown to contribute to tumor growth, metastasis, and resistance to therapy, the latter also in combination with Myeloid‐Derived Suppressive‐Like Cells [27], and where alveolar macrophages (AMs) have been increasingly recognized as the major subset of pro‐tumorigenic MPs [28].

Although smoking—as the major risk factor for lung cancer—has been linked with 2.5 million lung cancer deaths per year [29], a remarkable increase of cases in young individuals (especially females in Asian countries) not related to smoking has attracted attention in recent years [29]. This suggests that additional risk factors including diet and possible dietary exposures, for example, to BMMFs, need to be assessed in more detail to test their role as possible cancer drivers and to improve molecular understanding of indirect carcinogenesis as a basis for prevention and therapy. To assess presence of BMMF and a possible BMMF contribution to lung cancer development, we performed a multi‐omics BMMF detection based on IHC with anti‐Rep ABs in different sample collections of lung cancer patients (representative for Germany) as well as age‐matched controls and conducted bulk/sc‐RNAseq in silico BMMF profiling. A significantly increased population of BMMF‐positive M2‐like AMs was observed in peritumor tissues of cancer patients when compared to tumor and healthy controls, which supports a possible relevance of BMMFs as biomarker for lung cancer.

## 2 Materials and Methods

### 2.1 Patient Samples

Tissue samples were provided by the NCT tissue bank and Lung Biobank Heidelberg in accordance with the local regulations. A complete overview of the cohort (16 females, 31 males, 45–80 years of age, median age 63 years) is given in Table S1. Cryosamples were snap‐frozen within 30 min after resection and stored at −80°C. Detection of BMMF Rep expression was analyzed based on formalin‐fixed, paraffin‐embedded (FFPE) tissue sections from non‐cancer individuals (hamartoma patients, *n* = 19, no obvious diseases), tumor and paired peritumor tissues (taken at a distance of at least 2 cm from the tumor, patho‐histologically assessed as tumor‐free) of lung cancer patients (*n* = 34, each) as well as unpaired tumor tissues (*n* = 3) and unpaired peritumor tissues (*n* = 8). Cryo‐sections were obtained from non‐cancer individuals (hamartoma patients) (*n* = 19), tumor and paired peritumor tissues of lung cancer patients (*n* = 20, each).

### 2.2 Production and Characterization of New Mouse Monoclonal ABs

New mouse monoclonal ABs raised against the Rep of BMMF1 isolate C1HB.4 (CDS63405.1, 36 kDa) (AB 20 & 21) or Reps of H1MSB.2 (CDS63399.1, 37 kDa), C1MI.3M.1 (nt 292‐1260 of LR215499.1, 37 kDa) and C1MI.9M.1 (VEV85364.1, 38 kDa) (AB 22 & 23) were produced/characterized as described before [22], but by utilizing a parallel immunization with H1MSB.2, C1MI.3M.1, C1MI.9M.1 Rep to generate both cross‐detecting and antigen‐specific ABs. Antigen reactivity was tested by western blotting with HEK293TT (RRID: CVCL_1D85, authenticated) lysates after overexpression of Rep fusion‐proteins (ZsGreen1‐C1MI.9M.1 Rep [65 kDa, Clontech pZsGreen1C1, Mountain View, CA], NanoLuc‐H1MSB2 Rep [55 kDa, Promega pNLF1C, Madison, WI], 3×Flag‐C1MI3M.1 Rep [40 kDa, Invitrogen p3×FLAGCMV7.1, Carlsbad, CA]) and by enzyme‐linked immunosorbent assay (ELISA). For ELISA, in brief, 200 ng antigen was coated (96‐well Maxi Sorp, Thermo Scientific, Waltham, MA, in 100 μL 8 M urea pH 8.0, 4°C, overnight), blocked (PBS, 0.05% Tween20 [0.05% PBST], 10% BSA, 1 h, RT) and washed (5×, 0.05% PBST) before antibody addition (1 h incubation, RT) and a further five washing cycles. After incubation with secondary AB (rabbit anti‐mouse IgG HRP, 0.05% PBST, Rockland, 1 h, RT) and washing (3×), TMB substrate was added (TMB super slow, Sigma Aldrich, St. Louis, MO) and the colorimetric reaction was terminated with 1 M sulfuric acid prior to measurement with a plate reader (FLUOstar Omega, BMG Labtech, Ortenberg, Germany). Detailed systematic analyses of anti‐BMMF Rep ABs were performed via ELISA on different Rep and non‐Rep targets including all BMMF1 Rep ABs [30].

### 2.3 Tissue Analyses and Quantification

FFPE or fresh lung tissues obtained after surgery were used for the analysis. FFPE sections were prepared, processed for IHC‐detection (diaminobenzidine [DAB] and co‐immunofluorescence detection) and digitalized as described previously [23] using anti‐Rep ABs (AB 1, 2, 3, 7, 10, 11, 15) described in [22, 23] (deposited at DSMZ‐German Collection of Microorganisms and Cell‐Cultures), new ABs AB 20, 21, 22, 23, anti‐MARCO (Invitrogen, #PA5‐64134, rabbit IgG, polyclonal; 6 μg/mL), anti‐CD11b (Abcam, ab133357, rabbit IgG, monoclonal; 1.18 μg/mL), anti‐CD163 (Novus Biologicals, NB110‐40686, mouse IgG1, monoclonal; 5 μg/mL), and anti‐CD68 ABs (Cell Signaling Technology, #76437, rabbit IgG, monoclonal; 1:500 dilution). Cell‐based quantification of Rep^+^, CD68^+^, CD163^+^, MARCO^+^ and CD11b^+^ cells based on ImageJ/Fiji (RRID:SCR_002285 V1.53q, open source, approximately 4000 nuclei per tissue) was run as specified in [31] using ANOVA testing for significance and multiple testing for different cell populations (GraphPad Prism RRID:SCR_002798 V8, V9.3.0, GraphPad Software Inc., Boston, MA). The data was represented as % Rep‐, CD68‐, CD163‐, MARCO‐, CD11b‐positive cells and/or its combinations either normalized to the total number of nuclei or the number of specific macrophage populations.

### 2.4 Bulk RNA/DNA Sequencing and Single Cell RNA Sequencing

Available WGS and RNAseq data of lung cancer cohorts from the pan‐cancer analysis of whole genomes (PCAWG) [32] (WGS: 90 patients, RNAseq: 98 patients) as well as from a scRNA‐seq dataset [33] (30 patients) was employed for in silico screening of BMMF reads using the D‐ViSioN pipeline [34] as described before [35]. The resulting metadata for BMMF positivity was added to the Seurat Object [36] encapsulating the scRNA‐seq data (using RStudio V4.2.2, Posit PBC, Boston, MA, with Seurat V4.3.0 built‐in). Cells with at least one BMMF read were annotated as BMMF‐positive. M1‐/M2‐like macrophage phenotypes were assigned with two lists of marker genes [33] and M1/M2‐scores of BMMF‐positive and BMMF‐negative cells tested with RStudio built‐in robustbase (V0.95.0).

### 2.5 Preparation of Samples for Western Blotting

Pieces from frozen lung tissue (~100 mg) were dissected on ice and subjected to lysis in 300 μL RIPA buffer containing protease/phosphatase inhibitor (Sigma‐Aldrich, USA, Roche, Basel, Switzerland) by using tissue homogenization (Bertin instruments, Montigny‐le‐Bretonneux, France: 3 × 20 s interval, “hard”, 4°C) followed by clarification of the lysate via centrifugation (20 min, 22.000 g, 4°C). 25 μg of protein were subjected to 4%–20% SDS‐PAGE gels (Invitrogen, USA), and the gel transfer‐blotted on nitrocellulose (Turboblot, Biorad, Hercules, CA) for use in WB as described previously [22].

### 2.6 Immunoprecipitation (IP)

One peritumor tissue and one tissue adjacent to hamartoma with strong WB band detection were used for Rep IP. 20 μg protein (in 1 mL RIPA buffer) were incubated with each 2 μg of AB 2 or AB 10 for 1.5 h at 4°C before addition of 30 μL Protein A/G plus‐Agarose slurry and additional 1.5 h incubation. After washing (3× centrifugation at 600 g, 3 min, 4°C, RIPA buffer), elution was performed with 2.5‐fold Lämmli buffer, followed by SDS‐PAGE and immunodetection with AB 3. To test IP, WB positive bands were analyzed by mass spectroscopy.

### 2.7 Mass Spectroscopy

Proteins (20 μL) were run for 4 cm into an SDS‐PAGE. After Coomassie staining, target bands were cut and digested with trypsin [37] adapted to on a DigestPro MSi robotic system [INTAVIS Bioanalytical Instruments AG]. Dried peptide samples were reconstituted (97.4% water, 2.5% hexafluoro‐2‐propanol and 0.1% trifluoroacetic acid (TFA)) and half the resolubilization volume was used for LC–MS/MS analysis on an Ultimate 3000 UPLC system (Thermo Fisher Scientific, Waltham, MA) with an Orbitrap Exploris 480 mass spectrometer (60 min) and online desalting (Acclaim PepMap300 C18, 5 μm, 300 Å pore trapping cartridge; Thermo Fisher Scientific, Waltham, MA; 3 min, 30 μL/min flow of 0.05% TFA in water). The analytical multistep gradient (300 nL/min) was performed with a nanoEase MZ Peptide analytical column (300 Å, 1.7 μm, 75 μm x 200 mm, Waters) using solvent A (0.1% formic acid in water) and solvent B (0.1% formic acid in acetonitrile). Concentration of B was linearly ramped from 5% to 30% for 43 min, followed by a quick ramp to 80%. After 1 min, the concentration of B was lowered to 2% followed by equilibration (10 min).

Eluting peptides were analyzed in the mass spectrometer using data dependent acquisition (DDA) mode. A full scan at 60 k resolution (380–1400 m/z, 300% AGC target, 45 ms maxIT) was followed by up to 1.5 s of MS/MS scans. Peptide features were isolated with a window of 1.4 m/z, fragmented using 26% NCE. Fragment spectra were recorded at 15 k resolution (100% AGC target, 54 ms maxIT). Unassigned and singly‐charged eluting features were excluded from fragmentation and dynamic exclusion was set to 20 s.

Analysis was carried out by MaxQuant (1.6.14.0) [38] using an organism‐specific database extracted from Uniprot.org (human reference database, 79,038 unique entries from 3rd January 2022) and a custom sequencing‐based database (containing 4.489 entries) under default settings (except that label‐free quantification [LFQ, default settings]) and the iBAQ‐value generation were enabled [39].

### 2.8 Quantitative PCR

Quantitative PCR was performed with Power SYBR Green master mix on QuantStudio 5 System instrument (Thermo Fisher Scientific, Waltham, MA) with primers CMI5.240‐172 (5′‐GAACATAGAGCGAGCCGTGT, 3′‐AAACCACGCCAAACTCCTCC) according to the manufacturer's instructions. The PCR program consisted of an initial denaturation step at 95°C for 10 min, followed by 40 cycles of denaturation at 95°C for 15 s and annealing/extension at 60°C for 60 s. Gene expression levels were normalized to GAPDH (triplicate analysis), and ΔCT values were calculated as the difference between the CT values of the target gene and the housekeeping gene using QuantStudio Design & Analysis Software.

### 2.9 RNA In Situ Hybridization

FFPE tissue sections were deparaffinized using xylene and rehydrated (99, 96, 70% ethanol series and tris buffered saline), followed by peroxidase block (hydrogen peroxide incubation, 10 min, 21°C), antigen retrieval (95°C boiling in RNAscope Target Retrieval Reagent, 15 min, ACDBio, Newark, CA) and protease treatment (RNAscope Protease Plus, 30 min, 40°C) according to manufacturer's protocol. Hybridization was performed with RNAscope probes for 2 h at 40°C in a HybEZ Hybridization System (probes: C1HB.4—targeting bp 331–1606, H1MSB.2—targeting bp 580–1601, C1MI.3M.1—targeting bp 273–1352, H1MSB.1—targeting bp 461–1565; individual sense/anti‐sense probes). Slides were washed (RNAscope Wash Buffer), followed by signal amplification (RNAscope Signal Amplification Reagent) and detection using the RNAscope 2.5 HD Detection Reagent—BROWN (Advanced Cell Diagnostics Inc., Newark, CA, USA), including counterstaining with Gill's hematoxylin. Tissues were dehydrated, mounted (Eukitt Mounting Medium, Thermo Fisher Scientific, Waltham, MA) and scanned with a Hamamatsu scanner S60 (40× magnification, image adjustments with Hamamatsu NDP software, Shizuoka, Japan).

## 3 Results

### 3.1 Rep Expression is Congruent With CD68 ^+^ (Alveolar) Macrophages in Peritumor Tissue

**FIGURE 1:**
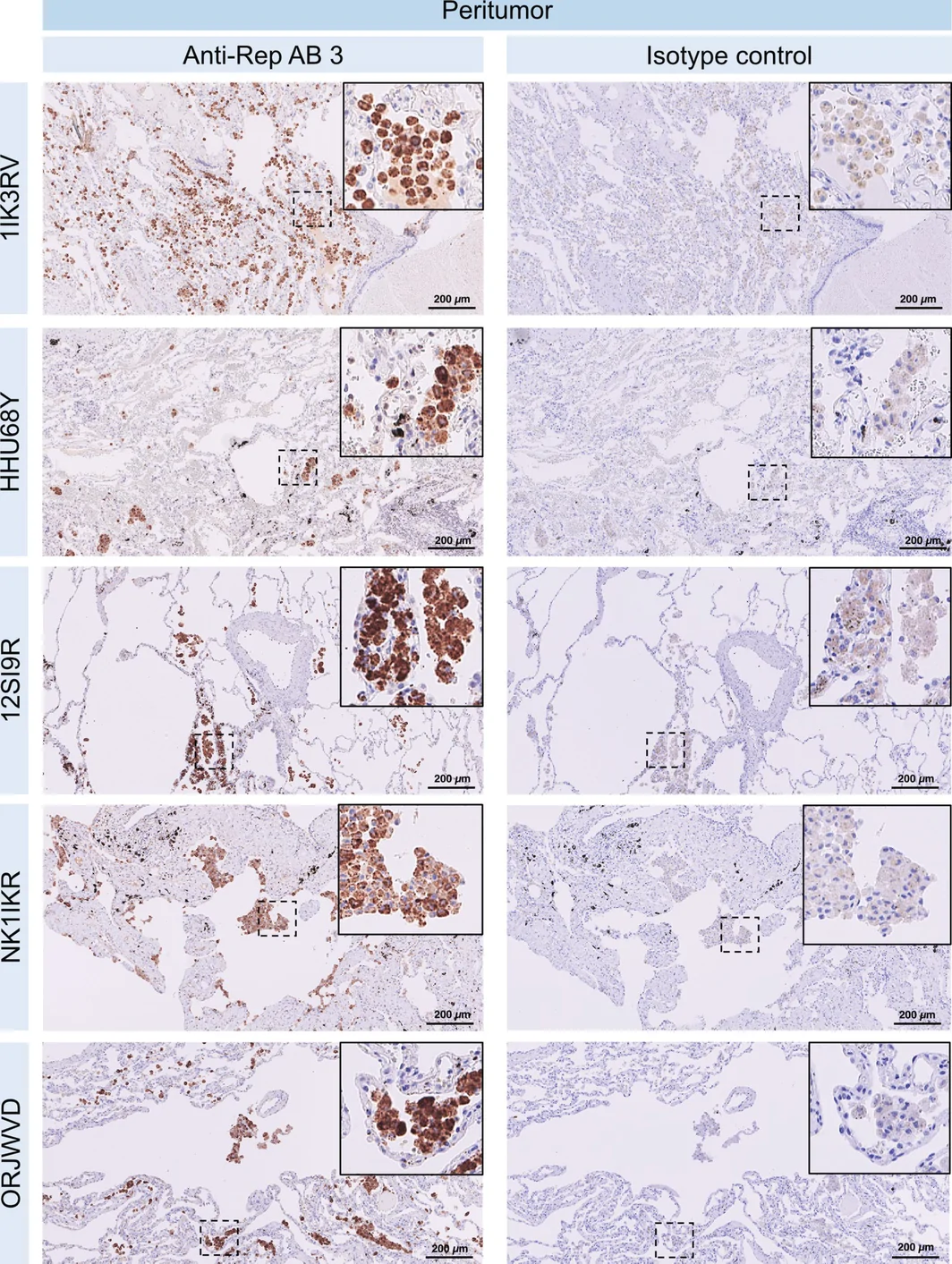
Immunohistochemical detection of BMMF Rep in lung cancer peritumor tissues. IHC DAB detection applied on consecutive, representative lung cancer peritumor (FFPE) tissue sections using anti‐BMMF Rep AB 3 or isotype control antibody (IgG1) (including 4‐fold enlargements of indicated tissue areas).

**FIGURE 2:**
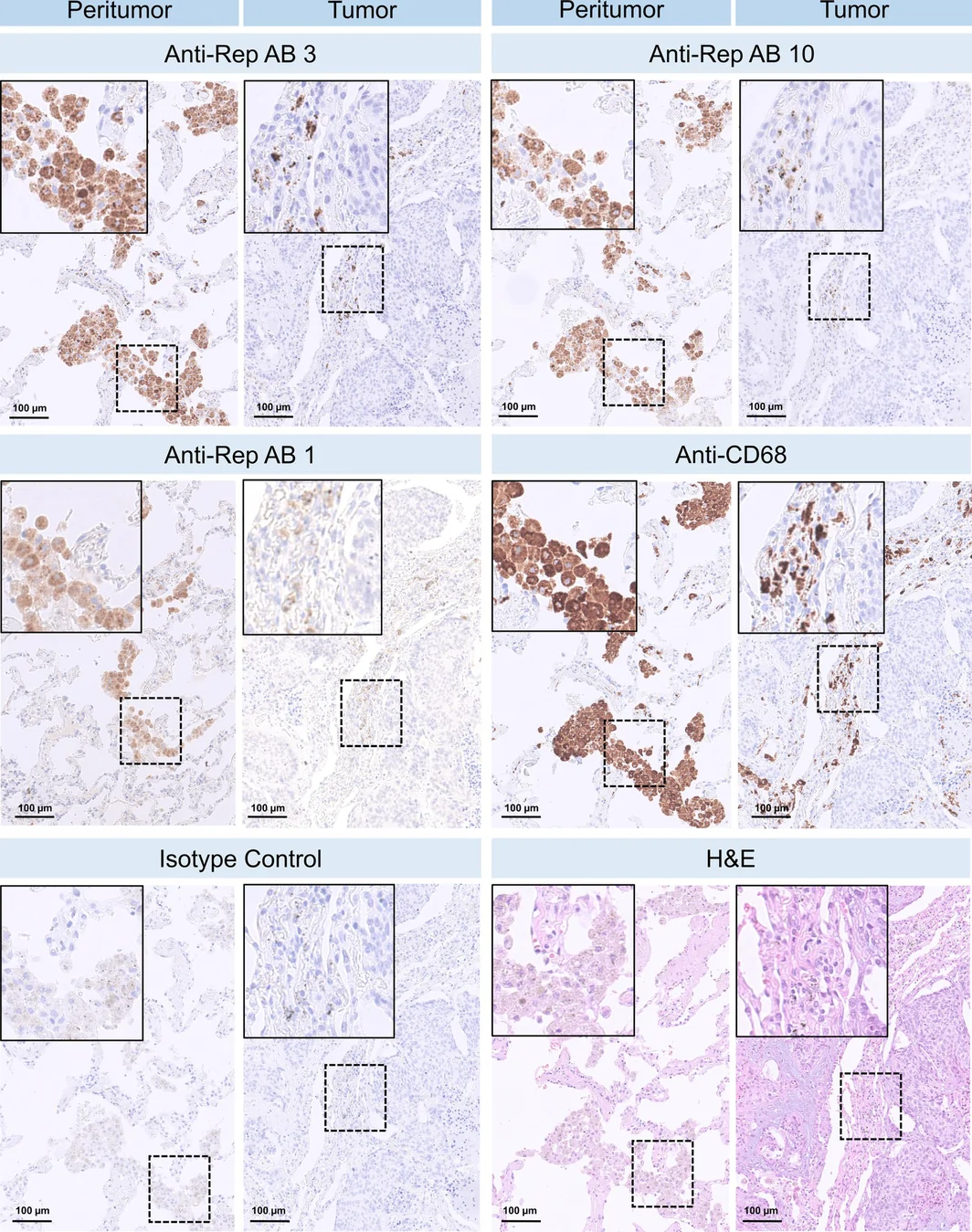
Immunohistochemical detection of BMMF Rep and CD68 in peritumor and tumor tissues. Consecutive (paired) peritumor and tumor tissue sections of a lung cancer patient were processed with anti‐BMMF Rep ABs AB 3, AB 10, AB 1, anti‐CD68 or isotype antibody (IgG1), including H&E control staining (2‐fold enlargements of indicated tissue areas). Note that for representation of tumor tissues, some of the very sparse tissue areas showing Rep expression were selected for representation of the otherwise predominantly negative tumor tissues (see IF quantifications for quantitative comparison).

We first characterized BMMF Rep expression in FFPE tissue cohorts comprising paired tumor and peritumor as well as tissues adjacent to hamartoma, based on (co‐) immunohistochemical DAB tissue detection with anti‐Rep ABs (cohort description in Table S1, patient information in Table S2). Tissues adjacent to hamartoma were used as non‐cancerous control samples (healthy). As observed before in a smaller study [35], Rep expression was detected predominantly in peritumor tissues (taken at least around 2 cm away from the tumor, =tumor‐free) (Figure 1). All peritumor tissues were tested positive (*n* = 42), while only 28% (10 out of 37) of tumor tissues revealed positive detection with less intensity and coverage (Figure 2). Figure 2 also depicts an example of the rare observation of existing, but weak Rep expression in a tumor region with a pronounced stromal compartment (and high macrophage number), which was usually not observed. Eight out of 8 different monoclonal anti‐Rep ABs with different epitopes revealed positive tissue detections. AB 3 and AB 10 showed strongest detections and highest numbers of positive tissues. AB 3 showed positive detection in 66 out of 97 tissue samples (11/36 tumor, 42/42 peritumor, 13/19 healthy). AB 10 revealed expressions in 47/93 tissues (6/37 tumor, 34/38 peritumor, 7/18 healthy) (representative expressions in Figure S1), followed by AB 21 (46/94 tissues; 4/34 tumor, 33/41 peritumor, 9/19 control), AB 1 (45/97 tissues; 3/36 tumor, 31/41 peritumor, 11/19 control) and AB 2 (40/92 tissues; 4/34 tumor, 32/38 peritumor, 4/15 control) (Table S3). No positive detection or detection with markedly less intensity was observed with isotype controls, but black‐colored tar deposits were present in most tissues (Figure 2). Competition of IHC detection with purified H1MSB.1 Rep protein resulted in significant reduction (up to 55%) of the tissue expression (Figure S2).

When testing the association of Rep detection with cell markers on consecutive tissue sections, Rep expression appeared as speckles predominantly in the cytoplasm of CD68‐positive enlarged and roundish macrophages (MPs) on lung alveolar surfaces (Figure 2, and Figure S3 for more donors). These cells were subsequently confirmed as MARCO^+^ alveolar MPs (Figure S4). Rep expression revealed a higher granularity compared to the more even distribution of CD68/MARCO. No or low Rep expression was observed in CD68^+^ but smaller interstitial MPs (Figure S3, red arrows), which were histologically defined via CD11b detection (Figure S4). A similar detection of cytoplasmic speckles on alveolar MPs was observed with additional anti‐Rep ABs AB 1/2/7/10/11/15 and the C1HB.4 Rep‐reactive AB 20 and 21 and H1MSB.2/C1MI.3M.1/C1MI.9M.1 Rep‐reactive AB 22 and AB 23 (Figure 2 for AB 1/3/10 and Figure S5 for the new AB 20–23 [see below]).

### 3.2 Increased Rep Expression in Peritumor Compared to Tumor and Control Tissues

**FIGURE 3:**
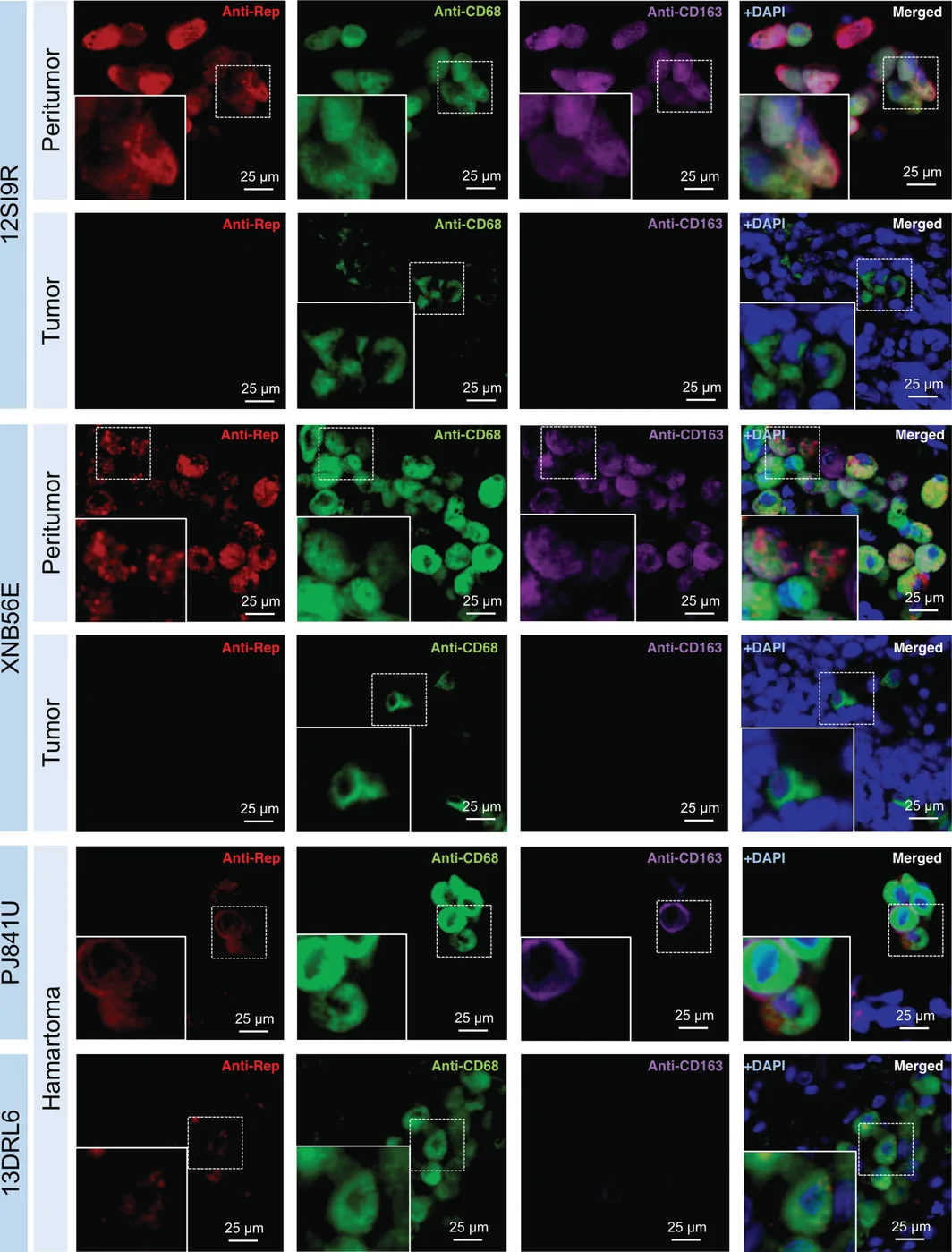
Co‐immunodetection of Rep, anti‐CD68 and anti‐CD163 expression in lung tissues. Tissue sections from 2 representative lung cancer patients (paired peritumor and tumor) and a control tissue (close to hamartoma) (in concordance with cell‐based quantification) processed via triple immunofluorescence microscopy with anti‐Rep (AB 3, red), anti‐CD68 (green) and anti‐CD163 (purple) antibodies, including DAPI nuclear staining (including 2‐fold magnification, scale bars: 25 μm).

To quantify the number of Rep‐positive cells, we applied co‐immunofluorescence microscopy with anti‐Rep (AB 3), anti‐CD68 and anti‐CD163 ABs (and MARCO and CD11b). Rep expression (red) localized together with CD68 (green) and CD163 (magenta) MPs in the peritumor, with less detection in control tissues and almost no detection in tumor tissues (Figure 3). The speckles‐like structures observed with the anti‐Rep detections (red) were not found in the CD68 (CD163) detection channels. As expected from the DAB data, co‐IF of Rep together with MARCO as a marker of alveolar MPs revealed a congruent staining, which was observed at a much lower level in parallel staining of Rep and interstitial MPs with CD11b in consecutive cuts (Figure S6A).

**FIGURE 4:**
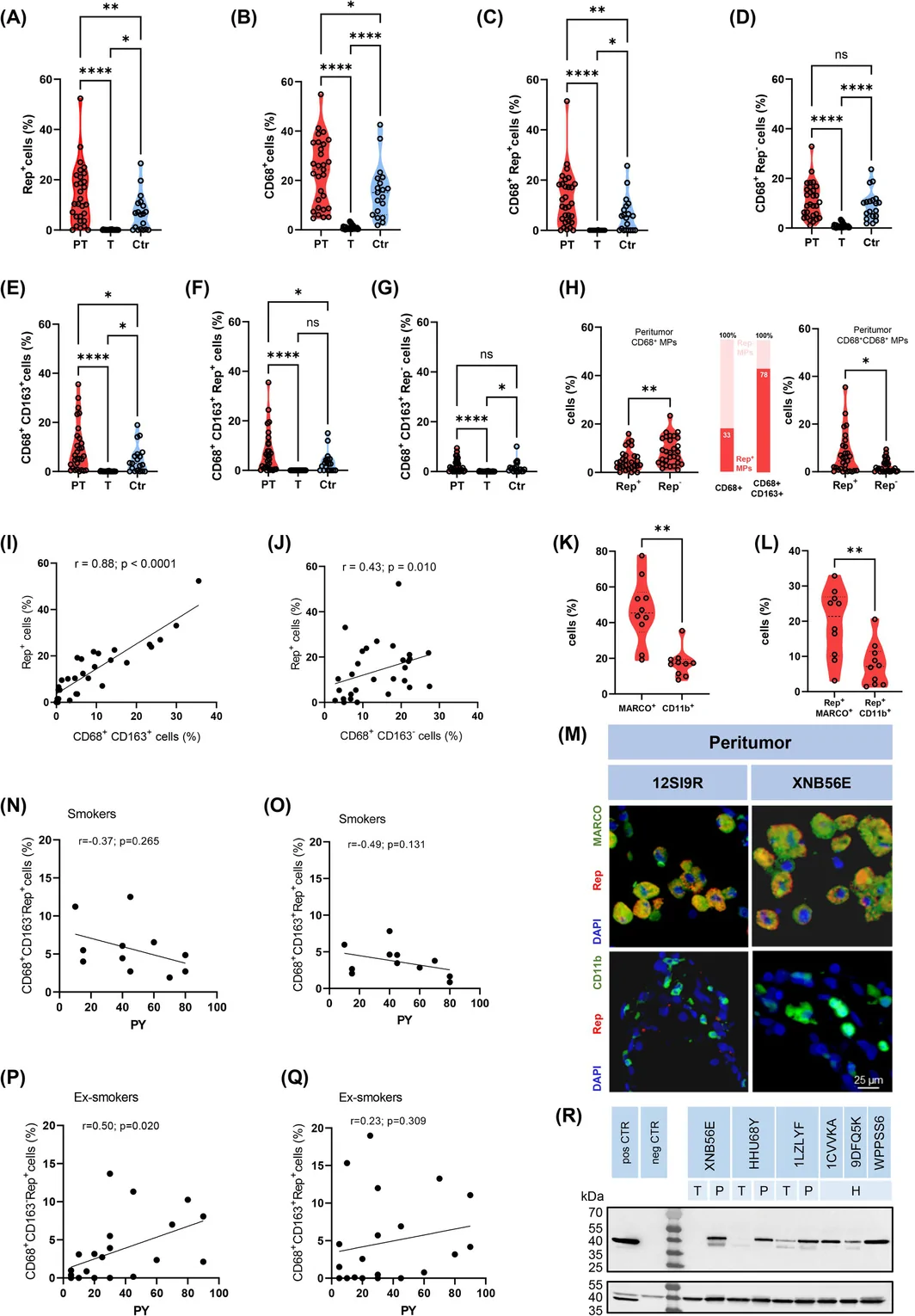
Quantification of Rep^+^ (A), CD68^+^ (B), Rep^+^CD68^+^ (C) and Rep‐CD68^+^ (D) cells in peritumor and tumor tissues (*n* = 29, each) of lung cancer patients as well as tissue adjacent to hamartoma (*n* = 19 individuals), based on co‐immunofluorescence microscopy. Quantification of CD68^+^CD163^+^ (E), Rep^+^CD68^+^CD163^+^ (F) and Rep^−^CD68^+^CD163^+^ cells (G) of the same cohorts. (H) Comparison of Rep^−^and Rep^+^ non‐M2‐like MPs (left) or M2‐like MPs (right, including bar‐chart indicating fractions of Rep^+^ versus Rep^−^ non‐M2 or M2‐like MPs normalized to non‐M2/M2 MP number in the center) (one‐way ANOVA test, *p < 0.05, **p < 0.01, ***p < 0.001, ****p < 0.0001). Correlation plots for CD68^+^CD163^+^ (I) and CD68^+^CD163^−^ (J) cells in peritumor tissues, plotted against the number of Rep^+^ cells in single patients. (K, L) Quantification of MARCO^+^ and CD11b^+^ cells (left) and Rep^+^MARCO^+^ and Rep^+^CD11b^+^ cells (right) in peritumoral tissues (*n* = 10 representative tissues). (M) Representative immunofluorescence images of peritumoral tissues stained for MARCO (upper row) or CD11b (lower row) (green) together with Rep (red) in two patients (12SI9R and XNB56E). Scale bar, 25 μm. Correlation plots for the number of Rep^+^CD68^+^CD163^−^ (N) and Rep^+^CD68^+^CD163^+^ (O) cells in peritumor tissues of actual smokers (*n* = 11), plotted against the intensity of smoking (pack‐years, PY) of the respective individual. Correlation plots for the number of Rep^+^CD68^+^CD163^−^ (P) and Rep^+^CD68^+^CD163^+^ (Q) cells in peritumor tissues of ex‐smokers (*n* = 21), plotted against PY (statistical test by simple linear regression and Spearman's correlation). (R) Immunodetection with anti‐Rep antibody AB 3 (top) and anti‐ß‐Actin (bottom) for representative tumor “T” and peritumor “P” tissue and lysates from tissues adjacent to hamartoma “H” (H1MSB.1 Rep‐overexpressing HEK293TT cell lysate used as positive control [target at 37 kDa], mock‐plasmid transfected cell lysate as negative control).

Cell‐based quantification confirmed a significant increase of Rep‐positive cells in peritumor (median 10.4% of all quantified cells) compared to the tumor tissues (< 0.1%, *p* < 0.0001) as well as compared to the hamartomas (6.5%, *p* = 0.009) (Figure 4, A). Quantification of CD68‐positive cells alone followed the same trend and was increased in peritumor (24.4%) when compared to tumor (0.5%, *p* < 0.0001) and hamartoma (15.1%, *p* = 0.021) (Figure 4, B). Importantly, while Rep‐negative MPs did not show a significant difference between peritumor and hamartoma, Rep^+^CD68^+^ MPs revealed a significant differentiation (peritumor: 10.1%, tumor: around 0%, hamartoma: 5.3%, *p* < 0.0001 and 0.009, respectively) comparable to Rep‐positive cells alone, indicating that CD68‐positive MPs represent the main population of Rep‐expressing cells in the lung tissues (Figure 4C,D).

### 3.3 Rep Expression Correlates With the Detection of (M2‐Like) MPs

We next differentiated MP populations into “M1‐like” (CD68^+^CD163^−^) or “M2‐like” (CD68^+^CD163^+^) cells. Generally, M2‐like MPs were significantly increased in the peritumor (6.3%) compared to the tumor (< 0.1%, *p* < 0.0001) and hamartoma (3.3%, *p* = 0.048) (Figure 4E). Almost identical trends were observed for Rep^+^ M2‐like MPs (peritumor: 4.6%, tumor: < 0.1%, hamartoma: 2.6%, *p* < 0.0001 and 0.044, respectively) (Figure 4F), while Rep‐negative M2 MPs did not show a significant difference between peritumor (1.3%) and hamartoma (0.8%, *p* = 0.552) (Figure 4G). Quantification of M1‐like MPs in general, or Rep^+^ and Rep^−^ M1‐like MPs did not result in any significant differences between peritumor and control groups (Figure S7).

In peritumor tissues, a higher number of cells were Rep‐negative M1‐like MPs (8.2%) when compared to Rep‐positive M1 MPs (4.0%, *p* = 0.0053) (Figure 4H, left). 67.2% of all M1‐like MPs were Rep‐negative, only 32.8% Rep‐positive. In contrast, there was a higher number of Rep‐positive M2‐like MPs (4.6%) compared to Rep‐negative M2 MPs (1.3%, *p* = 0.0163) (Figure 4H, right), with 78.3% of all M2‐like MPs being Rep‐positive and only 21.7% Rep‐negative, indicating a closer association of Rep detection with M2‐like MPs. This was also confirmed by a significant and stronger correlation of the number of Rep‐positive cells with CD68^+^CD163^+^ M2‐like MPs (*r* = 0.88, *p* < 0.0001) (Figure 4I) when compared to the association of Rep‐positive cells with CD68^+^CD163^−^ M1‐like MPs (*r* = 0.43, *p* = 0.010) (Figure 4J). Quantifications also showed an increased amount of alveolar (MARCO^+^, 45.4%) over interstitial (CD11b^+^, 17.1%, *p* = 0.002) MPs in (consecutive) peritumor tissues (Figure 4K). This was flanked by increased numbers of alveolar Rep^+^MARCO^+^ MPs (21.39%) versus interstitial Rep^+^CD11b^+^ cells (7.16%, *p* = 0.002) (Figure 4L) and a positive association of Rep expression with both markers, albeit at a different total level of Rep positivity (Figure S6B) (representative IF detections in Figure 4M, correlation data in Figure S6C,D).

Rep co‐detection with a marker of oxidative DNA damage (8OHdG, 8‐Hydroxydesoxyguanosin, red, Figure S8) revealed intense 8OHdG nuclear and, in part, cytoplasmic detection in Rep‐positive clusters of presumably alveolar MPs (yellow asterisks). Around and inside those clusters, strong (red) nuclear 8OHdG detection was also observed in other cells (red arrowheads), which faded with increasing distance (black arrowheads).

### 3.4 BMMF Expression is Higher in Low Dose Smokers

We stratified cell quantifications of the limited, available donor tissues by smoking status (“non‐smokers”, “ex‐smokers”, “smokers”) and found no significant differences between the groups (Figure S9A–D). All cohorts showed high variability in cell numbers, except smokers. Median numbers of Rep^+^, CD68^+^CD163^−^ M1, and CD68^+^CD163^+^ M2 cells were higher in non‐smokers (10.4%, 18.0%, 8.0%) and smokers (9.9%, 13.4%, 5.2%) than in ex‐smokers (6.6%, 10.1%, 4.9%), while Rep^+^CD68^+^CD163^+^ M2 cells were only increased in non‐smokers (6.5%) but low in ex‐smokers (2.6%) and smokers (3.1%). Ex‐smokers and smokers were further divided into “high” and “low” smoking groups (Figure S9E–H). Two observations stood out: (i) increased numbers of Rep^+^ cells in high‐dose ex‐smokers (12.2%) compared to low‐dose smokers (2.7%), albeit with similar M1 MP numbers (3.5% vs. 4.8%); (ii) slightly higher M1‐like MP numbers in low‐dose smokers (11.7%) compared to high‐dose smokers (3.5%). Partition according to gender showed no further differences (Figure S9I–N).

Correlation analysis of the quantity of positive cells with smoking intensity (pack‐years, PY) in smokers revealed a non‐significant trend for increased Rep^+^ cells with low PY (*r* = 0.38, *p* = 0.246). Comparable associations were observed for the numbers of M1‐ and M2‐like MPs, which were higher with lower smoking intensities (*r* = 0.56, *p* = 0.077 and *r* = 0.70, *p* = 0.020, respectively) (Figure S10A–C for smokers; D–F for ex‐smokers). Stratifying Rep^+^CD68^+^CD163^+^ cells reduced *p* values for anti‐correlation with smoking (*r* = 0.49, *p* = 0.131) (Figure 4N,O). In ex‐smokers, these anti‐correlative trends were reverted, especially for cancer patients with low PY, where CD86^+^CD163^+^Rep^+^ M2‐like cells showed heterogeneity, splitting into low and high responders (*r* = 0.23, *p* = 0.309) (Figure 4P,Q).

### 3.5 No Association of Rep Detection With Other Clinical‐Epidemiological Discriminators

Stratification of the cohort into male and females did not result in any significant differences between identified Rep‐positive cell populations in peritumor and control tissues (Figure S11A). Concerning age, a slight trend for increased detection of Rep^+^ and CD68^+^ cells with advanced age was observed, which did not depend on sex (Figure S11B–D). When investigating the possible association between Rep expression and lung pathology, no significant differences of Rep expression were found between non‐small cell lung cancer (NSCLC) and small cell lung cancer (SCLC), but there was a slight trend for increased Rep expression in SCLC, while NSCLC presented more heterogeneity and a stronger discrepancy of high and low responders (Figure S11E). This might indicate possible sub‐classes in NSCLC with different levels of association with BMMF detection.

### 3.6 Detection of BMMF Proteins in Lung Cancer Tissue Lysates by Immunodetection, IP and Mass Spectroscopy

Characterization of BMMF protein expression in lung cancer tissues (and controls) was additionally assessed by immunodetection, IP, and mass spectroscopy. Detection in paired tumor and peritumor tissues of 20 patients as well as 19 controls based on immunoblotting with AB 3 and AB 10 resulted in observation of bands at 37 kDa (occasionally as double bands) and slightly stronger ones at 45 kDa (Figure 4R and Figure S12). While all peritumor tissues revealed bands, 10 out of 20 tumor lysates lacked band detections. All but two hamartoma tissues revealed bands, but generally with less intensity, especially for the band at 37 kDa. On the whole, band intensities appeared slightly increased in peritumor samples compared to tumor and hamartoma samples (the 37 kDa band appeared more specifically associated with peritumor detection), were reproduced by detection with AB 3 (Figure S13A), and remained at the identical position in “spike in” experiments with purified H1MSB.1 Rep (Figure S13B). Blocking of anti‐Rep antibody detection by incubation of the WB membrane with purified H1MSB.1 Rep resulted in significantly decreased band detection (Figure S13C).

Since the target bands were located in protein‐rich regions of the gels, mass spectrometry for BMMF peptide identification included an enrichment using IP. Tissue lysates were subjected to IP with anti‐Rep ABs AB 10 and AB 2. Upon SDS‐PAGE and immunoblotting, observable bands at 37 kDa (Figure S13D) led to mass spectroscopy detection of a BMMF Rep fragment (LEEQFTQYDIEQISGLSSAYAVR) matching the Rep sequence of H1MSB.1, C1MI.2, C1MI.3M.1, C1MI.4, C1MI.5.1, C1MI.5.2, Sphinx1.76, and 25BAMI and localized between the winged‐helix 1 (WH1) and WH2 Rep domain (H1MSB.1 Rep aa 124‐147 [of CDS63398.1]) (Figure S13E).

### 3.7 Detection of BMMF Reads in Cancer Sequencings Supports BMMF Targets Previously Identified in the Wet Lab, but Also Hints at Additional BMMF Targets

**FIGURE 5:**
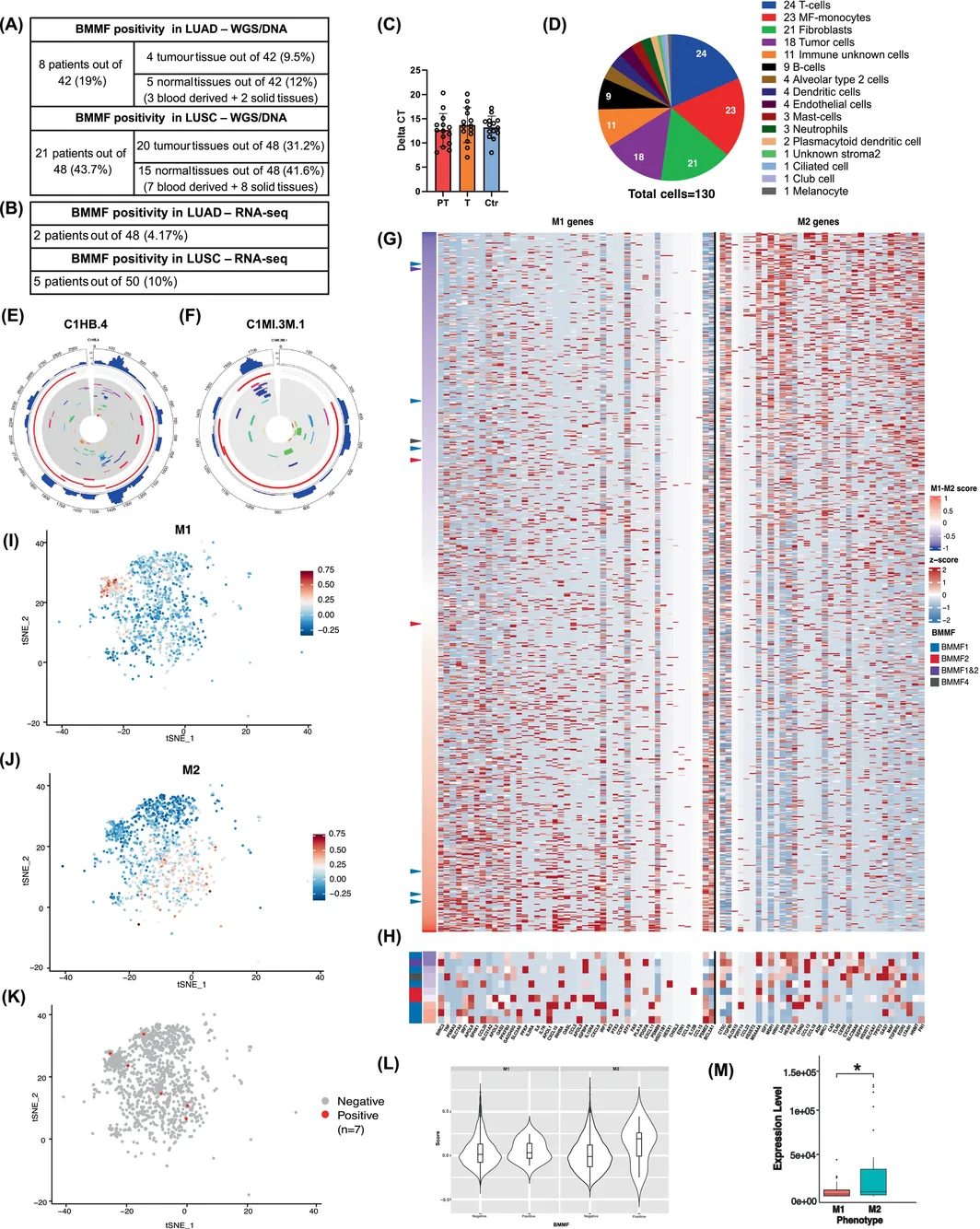
(A) Detection of BMMF reads in lung adenocarcinoma and lung squamous cell carcinoma from the WGS/DNA PCAWG dataset. (B) Detection of BMMF reads in lung adenocarcinoma and lung squamous cell carcinoma from the RNAseq PCAWG dataset. (C) RT‐qPCR with 14 paired tumor/peritumor tissues and 14 tissues from non‐cancer individuals, based on BMMF1 primer CMI5.240_172. Delta CT calculated by subtracting the CT for GAPD (statistical test by one‐way ANOVA). (D) BMMF reads detected in publicly available scRNA‐seq dataset. Out of 23,261 cells, 130 were positive for BMMF reads, with T‐cells and MPs as the most positive cells. Circular plots highlighting identified BMMF reads (blue outer circle) for the BMMF1 representatives C1HB.4 (E) and C1MI.3M.1 (F); ORFs marked in red outer circles; individual cells with BMMF reads are represented by different colors in the inner circle. (G) Heatmap for expression (gene‐wise Z‐score) of the selected M1‐ and M2‐associated genes in all lung MF‐monocytes (arrows on the left indicate BMMF‐positive cells) or in BMMF‐positive cells, only (H). Cells are ordered by the M1–M2 score (vertical ordinate). M1‐ or M2‐genes are ordered by the median expression across cells (horizontal ordinate). Color code for BMMF subtypes in positive cells—BMMF1: Blue; BMMF1&2: Violet, BMMF2: Red, BMMF4: Gray. (I, J) Differentiation into M1‐ or M2‐like MPs was done using a gene signature approach. (K) tSNE plot showing BMMF1‐positive MPs. (L) Robust linear regression models were fitted to assess the relationship between BMMF positivity and scores for M1‐ or M2‐like differentiation (p = 0.062). (M) Comparison between M1‐ and M2‐like gene expression levels of BMMF‐positive MPs in CD45^+^CD68^+^ sorted cells by bulk RNAseq (Mann–Whitney *U* test, *p* = 0.03).

In silico screening for BMMF reads in available WGS and RNAseq data of the lung cancer cohorts from the PCAWG project [32] resulted in identification of 240 BMMF reads. In WGS/DNA data (paired tumor/distant tissues), 8 of 42 lung adenocarcinoma (LUAD) patients (19%) revealed BMMF reads (4 positive tumors, 5 positive tumor‐distant tissues) (Figure 5A). Twenty‐one out of 48 lung squamous cell carcinoma (LUSC) patients (44%) showed BMMF reads (20 positive tumors, 15 positive distant tissues). In RNAseq data (only tumor), 2 out of 48 LUAD patients (4%) and 5 out of 50 LUSC patients (10%) revealed BMMF reads (Figure 5B). The identified reads covered C1HB.4, H1MSB.2, C1MI.3 M.1 and H1MSB.1 BMMF isolates (full list in Table S4), supporting the prior identification of BMMF1 Rep proteins based on detection with anti‐H1MSBI.1 Rep ABs. Upon the in silico identification, C1HB.4 Rep‐ and C1MI.3M.1/H1MSB.2 Rep‐detecting ABs were generated and included in the IHC analyses of lung cancer tissues and revealed a similar detection level of Rep^+^ alveolar peritumor MPs (Figure S5). In addition, RNA in situ hybridization using H1MSB.1 and H1MSB.2 sense‐ and anti‐sense‐specific probes showed positive speckles in the cytoplasm of AMs in peritumor tissues (Figure S14). RT‐qPCR detection of RNA prepared from a subset of fresh tissues, however, generated CT values of < 30 only for some of the samples and no noticeable differences between tumor, peritumor and control groups (Figure 5C).

### 3.8 BMMF‐Positive Cells Identified via scRNAseq Show Trend Toward a Unique Differentiation

To support in silico BMMF detection in LUAD and any associations of BMMF detection with M2‐like MPs predominantly identified via CD68/CD163 IHC, screening of lung cancer scRNAseq data for BMMF reads was performed, revealing BMMF‐positive cells in 25 out of 30 patients (83%). T‐cells and MPs represented the cell types with the highest numbers of BMMF reads, followed by fibroblasts and tumor cells (Figure 5D). Most of the BMMF hits were attributed to the BMMF1 group, particularly to the isolates C1HB.4 (Figure 5E) and C1MI.3M.1 (Figure 5F), which revealed a broad read coverage (full table of covered BMMF targets in Table S5, distribution of reads to BMMF groups 1–4 and sampling site in Figure S15). When considering only hits for BMMF1, MPs represented the most frequently covered/detected cell type with BMMF reads (14 cells in total).

To test a possible association of BMMF‐positive cells with M1‐ or M2‐like macrophage differentiation, lung MPs were first represented in a heatmap with gene‐wise Z‐scores for expression of M1‐ or M2 marker genes [33] (Figure 5G), arranged via the M1–M2 score (vertical ordinate) and median gene expression either over all cells (horizontal ordinate) or for BMMF‐positive cells exclusively (Figure 5H). Based on this, MPs were also grouped via 2D mapping into M1‐ (Figure 5I) or M2‐like (Figure 5J) MPs [33]. BMMF1‐positive cells (representing the target population in this study) were predominantly identified either between M1‐ and M2‐cell clusters or near M2‐like MPs (Figure 5K). This was supported by linear regression models confirming a closer association with M2‐like MP differentiation (M2: *p* = 0.062, M1: *p* = 0.900) (Figure 5L), and in silico analysis of CD45^+^CD68^+^‐sorted cells in bulk RNAseq, where BMMF^+^ cells showed stronger association with an M2‐like phenotype compared to M1 (*p* = 0.030) (Figure 5M) (markers listed in Table S6).

## 4 Discussion

### 4.1 Do BMMF Rep‐Positive AMs Represent Biomarkers for Lung Cancer?

**FIGURE 6:**
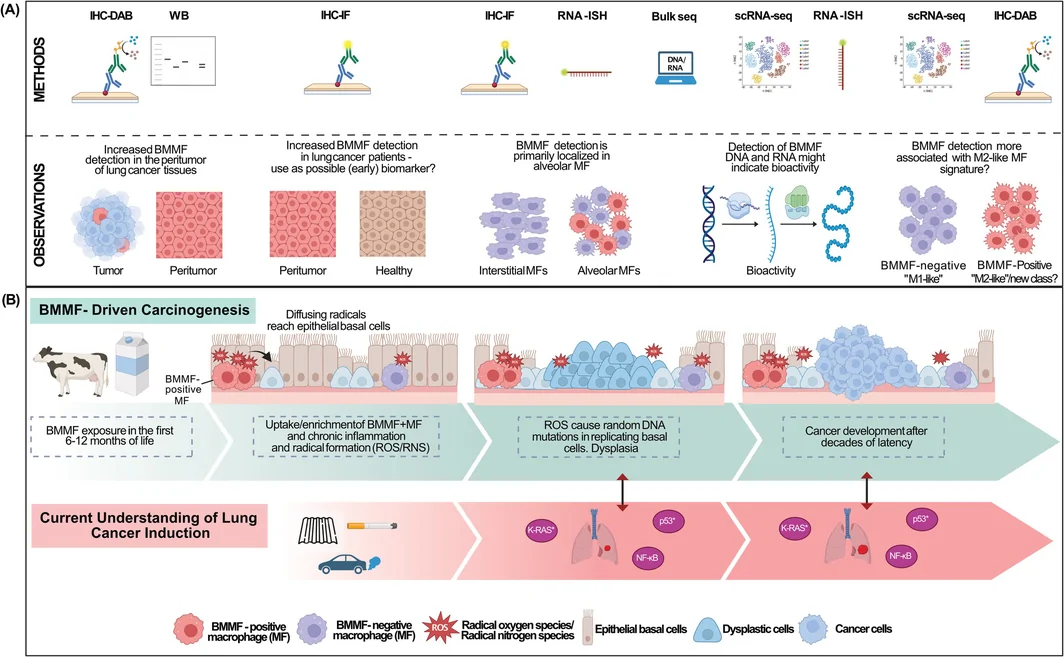
Proposed model for involvement of BMMFs as indirect driver of lung cancer (modified from [4, 8]). (A) In this study, Rep expression was observed in CD68^+^ AMs present specifically in the peritumor (IHC‐DAB and WB). Significantly increased numbers of Rep^+^CD68^+^CD163^+^ MPs were identified in the peritumor when compared to tumor and control tissues (IHC‐IF). BMMF detection was increased in AMs with a predominantly M2‐like differentiation (IHC‐IF, scRNA‐seq), supporting that BMMFs might contribute to lung cancer upon induction of pro‐tumorigenic macrophage populations, which further confirms their relevance as myeloid immune checkpoint for prevention and therapy. (B) Occupational and environmental exposures, pollution, and mostly cigarette smoking are the major risk factors for lung cancer development, inducing mutations in specific key regulators like K‐Ras, p53 and NF‐κB. The increase of lung cancer incidence in non‐smokers (specifically butchers and animal farmers) points toward the existence of additional risk factors. Our model of indirect carcinogenesis focuses on BMMFs as cancer risk factors contributing to induction of lung cancer. BMMF‐containing diet leads to the accumulation of BMMF‐positive AMs, which contributes to chronic inflammation, production of diffusing ROS/RNS and results in oxidative stress and an increased risk for DNA mutation, which occasionally targets cancer driver genes in neighboring, replicating basal cells. The latter might fix cancer driver mutations, which change into precursor cells for dysplasia and cancer after several decades.

Smoking represents the strongest risk factor for lung cancer development. Still, observation of increased lung cancer risk in individuals who have never smoked—especially younger females in Asia—in countries with a western or manifesting western diet prompts the search for additional cancer risk factors related to diet [40, 41]. Although our study focused on BMMF detection in a representative German lung cancer patient cohort with older age of the patients (63 years age average) and intrinsically lacking larger numbers of young lung cancer patients, we identified significantly increased expression of BMMF Rep as speckles in the cytoplasm of peritumoral AMs compared to controls, using immunohistology with anti‐Rep ABs (Figure 6A). Histological detection was supported by BMMF protein band detection via immunoblotting and IP. Following anti‐Rep IP, a Rep‐specific peptide was detected by mass spectroscopy. The peptide mapped to the H1MSB.1 Rep, which is the main target of the applied anti‐Rep ABs AB 3 and AB 10, but also of AB 1, 2, 7, 11, 15, which supports histologic detection. The 8 IHC‐positive ABs cover at least 5 individual epitopes, suggesting specific detection of BMMF proteins. Experiments to block antibody‐based detection with purified H1MSB.1 Rep resulted in significantly but not completely reduced detection levels on IHC and WB readouts, suggesting that in addition to H1MSB.1 Rep, other BMMF1 Reps with affinity to the ABs might exist. This assumption was confirmed by IHC tissue analyses with newly generated ABs reactive against the in silico genomics‐identified BMMF1 targets C1HB.4 (histologic Rep detection with AB 20 and 21) and H1MSB.2 as well as C1MI.3M.1 and C1MI.9M.1 (Rep detection with AB 22 and 23).

Semi‐automated quantification of positive cells after anti‐BMMF antibody detection revealed increased and strong expression of BMMF Rep predominantly in cytoplasmic speckles in more roundish and larger MPs, presumably MARCO^+^ AMs, of peritumor tissues in lung cancer patients compared to tumor parts and tissues adjacent to hamartomas of an age‐matched control group. Given the benign status of the hamartomas in the control group, the increased detection of Rep‐positive cells (10.4% vs. 6.5%, *p* = 0.009) and Rep^+^CD68^+^ (10.1% vs. 5.3%, *p* = 0.009) cells in the cancer group might represent a high‐risk cancer signature. These results match with the observation of increased Rep expression in the peritumor of CRC patients when compared to healthy controls [22, 23]. Still, in CRC peritumor, the number of MPs was comparable in case and control cohorts (about 35%–50% of interstitial cells were MPs). In contrast, in LUAD peritumor tissues, the levels of both CD68^+^ MPs and Rep^+^CD68^+^ MPs were increased in case versus control, while the numbers of Rep^‐^ MPs appeared invariant. This implies that only Rep^+^ MPs might represent a case–control‐specific and potentially lung cancer‐associated population of cells, but also that both cases and controls feature a high level of (Rep^‐^) MPs, which do not seem to be associated with disease status and might be reflected predominantly in CD11b‐positive interstitial MPs, which did not reveal BMMF expression.

Although the amount of MPs can vary across cancer types and between individual patients due to intra‐ and inter‐patient heterogeneities, the total proportion of MPs observed in our study aligns with previously reported MP densities in similar peritumor tissue contexts, with an overall average of approximately 130 TAMs per 1000 cells (or 13%); however, we identified a slight excess of CD68^+^ CD163^‐^ M1‐like MPs in comparison to CD68^+^ CD163^+^ M2‐like MPs in the peritumor, which might contrast with the excess of M2‐like TAMs observed (though for tumor tissues), for example, by Zheng et al. [42].

As the peritumor parts of the tissues are the last remnants of any ‘pre‐tumor’‐like tissue left after the tumor formed, they best reflect the conditions occurring during tumor formation, despite all limitations (e.g., tumor‐specific influence on the peritumor as part of the tumor microenvironment). Therefore, BMMF detection in peritumor might serve as a biomarker for lung cancer in general and might also open up the perspective on a potential causal contribution to tumor formation via indirect carcinogenesis: BMMF‐associated increased tissue inflammation, radical formation and induction of DNA damage (note the 8OHdG detection as surrogate marker for oxidative DNA damage) in neighboring, replicating basal cells (Figure 6B).

In general, the lower number of CD163^+^ MPs in tumor tissues observed in our study (IF and quantification) contradicts the more commonly reported enrichment [43] and might be due to a bias toward tumor nests (containing less MPs), but is still in line with other studies reporting on heterogenous sizes of CD163^+^ MPs populations [44] (and suggesting a link of low CD163^+^ MPs numbers with longer progression‐free survival). Reasons for heterogeneity might be differences in cancer subtypes, tissue quality and purity, and different ratios of tumor nests versus stromal tissue areas likely occurring during quantifications.

### 4.2 BMMF Rep Expression Correlates With the Number of CD68 ^+^ MPs

If BMMF expression is involved in induction of a Rep‐positive population of inflammation‐linked MPs, the number of Rep‐positive cells should be associated with the number of MPs. Indeed, correlation analyses revealed an association of the number of Rep‐expressing cells with the number of CD68‐positive cells. The strongest association of Rep‐positive cells was observed with CD68^+^CD163^+^ MPs (and MARCO‐positive presumably alveolar MPs), while a weaker association was observed with CD68^+^CD163^−^ MPs (and also some lower numbers of CD11b interstitial MPs), which again indicates BMMF presence predominantly in a specific niche/sub‐population of MPs, further supported by scRNA‐seq. However, peritumor tissues might still contain significant populations of recruited and repolarized CD68^+^CD163^+^ MPs of non‐alveolar type, which we did not stratify in this study in more detail.

### 4.3 Are BMMF Rep‐Positive M2‐Like MPs Drivers of Non‐Smoker‐Associated Lung Cancer Development?

Although a high number of M1‐like MPs was found in lung cancer peritumor tissues, which was not observed in CRC peritumor tissues, only M2‐like MPs show a case versus control difference in cell numbers in lung cancer. This discrimination intensifies when including Rep‐positivity. This data might generally suggest that M2‐like MPs could act as a pro‐tumorigenic population of immune cells, which is in line with literature [45]. CD163 remains one of the most reliable and widely used markers for identification of pro‐tumorigenic and therapy predicting TAMs in lung cancer [27, 42]. Therefore, in addition to therapy with common immune checkpoint inhibitors, which resulted in a marked but still limited improvement of 5 years survival from < 5 to about 13% in late‐stage NSCLC [46], approaches identifying and targeting M2‐like MPs—which might possibly represent a myeloid‐like immune checkpoint—have been discussed and developed [47]. Clodronate liposomes have been shown to effectively reduce TAM and inhibit lung tumor growth in mouse models [48]. Further, epigenetic modulators such as HDAC2 (overexpressed in M2 TAMs) are studied for targeting cancers with high M2‐like macrophage infiltration [49].

Still, M2‐like MPs also fulfill relevant protective functions in the tissue, such as anti‐inflammatory tissue modulation after acute inflammation and induction of tissue repair mechanisms [50]. Thus, elimination of all M2 MPs might not be effective to prevent or treat cancer, which might restrict current M2 MP‐based therapies, especially in the clinical context [51, 52]. Also, the role of TAMs in cancer development or how TAMs acquire their polarization is poorly understood and might also be associated with other factors, for example, air pollutants [53]. To identify, which M2‐like MPs might represent cancer‐promoting high‐risk cell populations (and putative targets for therapy), stratification into BMMF‐positive and negative MP populations might be crucial.

BMMF detection in lung (cancer) tissues and in the colon and pancreas indicates BMMF presence in organs with high blood turnover and might suggest BMMF transport and broad distribution via the blood circulation. BMMF exposure in the very early life (after birth/early weaning) might promote an inadequate and non‐effective immune response unable to clear BMMFs [8]. This might explain why increased consumption of dairy products during infant age (< 8 years, 4374 participants) results in significantly increased risk of CRC (OR = 4.31 (95% CI: 1.30, 14.22), *p* = 0.007) [54]. Concerning lung cancer, the same study identified a slightly increased lung cancer risk for low dairy uptake rates and decreased risk for high uptake rates, with the latter data being strongly confounded by the (unknown) smoking status in adult life. Lactose intolerance was reported to be associated with a lower lung cancer incidence [6, 8]. General support for involvement of chronic inflammation in lung cancer development originates from the observation of reduced cancer incidence upon regular use of NSAIDs [5]. Later life meat exposure (including exposure for example for meat workers) might increase the uptake and persistence of BMMFs especially in individuals already primed for increased BMMF uptake and susceptibility by early age BMMF exposure, the latter previously suggested to induce a dampened immune reaction and clearance of BMMFs later. However, because early and late age effects are heterogenous and influenced by many confounders such as smoking, clear conclusions on diagnostic or casual biomarker functions are still impossible now.

### 4.4 Heterogeneous Distributions of M1‐ and M2‐Like MPs in Lung Cancer Complicate Data Interpretation in the Context of Smoking

Cell‐based quantifications of MPs in general or M1‐ or M2‐like MPs in particular did not reveal significant differences with respect to the smoking status when grouped into non‐smokers, ex‐smokers or smokers. When including information about smoking intensity, no significant trends were observed either, with the exception of trends for low numbers of M1‐ (and M2) MPs in heavy smokers, but increased M1 (and M2) MPs in low‐dose smokers. This might reflect immunologic exhaustion in high‐dose smokers, in line with previous reports that smoking generally induces an increased number of neutrophils and MPs [55] until—in very heavy smokers—a state of immune exhaustion or tolerance and less immune cells is reached [56]. The general absence of differences in M1‐like MP numbers between peritumor tissue and healthy controls arguing against their use as cancer markers (despite predicting longer survival), [43] might be also explainable by the diverse smoking levels of the individuals fueling heterogenous populations sizes of M1‐like MPs.

For ex‐smokers, the situation is even less clear and confounded by the very frequent lack of information about how long before diagnosis individuals have quit smoking. Still, the number of Rep‐positive cells was increased in high‐dose ex‐smokers, which might be linked with a general deficit in M1 MPs that might clear BMMFs. Still, on the whole, non‐smokers revealed slightly higher detection levels of Rep^+^, Rep^+^ M1 MPs and Rep^+^ M2 cells compared to smokers and ex‐smokers. This tendency is also supported by the association of higher numbers of Rep^+^ and Rep^+^ M2 cells with lower PY in correlation plots for smokers, which is reversed for ex‐smokers. These observations might indicate that especially in lung cancer patients with low smoking status, BMMFs might represent an alternative cancer risk factor; however, the cohort sizes of the test groups in this exploratory study were too small to draw any more consistent conclusions.

### 4.5 Detection of BMMF Reads in Bulk and scRNAseq

Remarkably, despite the relatively low sequencing depth of individual cells in the scRNA‐seq data (mean sequencing depth = 5 × 10^8^ bases per cell) in comparison to bulk RNA‐seq datasets, 0.6% of all cells (130 out of 23,261) were BMMF positive. In line with the detections with anti‐BMMF ABs, the most frequently detected isolates included the H1MSB.1 isolate covered by previous immunohistochemical analyses but also additional targets, such as C1HB.4 and C1MI.3M.1, whose Rep proteins were detectable with the newly generated ABs. The relative accumulation of BMMF‐reads in cells of the immune system supports the notion that BMMFs stimulate a reaction of the immune system, thus providing another piece of evidence for the indirect carcinogenesis via inflammation hypothesis. Still, the high CTs observed by RT‐qPCR and a low number of positive cells in scRNA‐seq generally argue against a broad bioactive transcription of BMMFs in tissues of lung cancer patients. However, the observation of intense ISH detection in peritumor alveolar MPs (especially with H1MSB.1‐ and H1MSB.2‐Rep gene‐specific anti‐sense probes) indicated the potential existence of a cellular niche that allows BMMF transcription. The long half‐life time of alveolar MPs of several weeks to months might contribute to transcription and allow BMMF induced reprogramming of the cells into the observed differentiation patterns, which did neither associate with M1 nor clearly with M2 MPs. Alternatively, given the objectively low BMMF detection in RNA and DNA data, the BMMF protein itself (Rep) could act as a potential pathogen; a transmission and bioactive function of Rep proteins similar to BMMF Reps have been already shown in vitro, [57]. In summary, this BMMF‐driven hypothesis could explain the so far unresolved existence of a specific and pro‐tumorigenic population of myeloid cells contributing to a myeloid immune checkpoint, and might provide relevant targets for prevention and therapy.

### 4.6 Translational Relevance

Although smoking and other environmental factors are known to increase the risk of lung cancer, we currently lack understanding of the molecular mode of action of risk factors like diet and consumption of red meat in relation to lung cancer, especially in non‐smoking‐associated cases, which are increasing steadily. Here, we found that detection of proteins encoded by a group of plasmids termed BMMFs—originally identified in bovine meat and milk—is associated with inflammatory immune cells and increased in tumor‐adjacent tissues of lung cancer patients. This indicates a role as biomarker for lung cancer, which might also include a causal contribution. BMMF detection, for example, in lung biopsies, breath condensate and blood tests for early cancer detection, might help to identify individuals at high cancer risk, who require, for example, tighter surveillance and changes in dietary habits to improve options for primary and secondary prevention.

### 4.7 Limitations of the Study

Detection of BMMF DNA and RNA was limited to sensitive ISH, or scRNA‐seq/NGS/RNAseq, with low numbers of positive cells not allowing detailed transcriptional analyses. Expansion of the data analyses with biomaterial from different geographical locations, involving data on patient survival, serum/blood, sputum and breath condensate, and generally more samples to resolve any possible inverse association with smoking, as well as large‐scale genomics would be relevant to further support the possible association of BMMFs with lung cancer and to generate more sensitive tools for detection that could be used for preventive and therapeutic intervention.

This current study cannot provide molecular‐pathological conclusions or proof‐of‐concept for any causal or pathogenic involvement of BMMFs in carcinogenesis, which must be assessed with prospective screening setups (e.g., serology) and/or models to test BMMF‐specific tumor induction, for example, by utilizing/analyzing BMMF‐exposure in cell/organoid or mouse models.

## Author Contributions

**Nives Cecere:** conceptualization, investigation, writing – original draft, validation, data curation, formal analysis, writing – review and editing, software, visualization. **Kristina Alikhanyan:** conceptualization, investigation, writing – review and editing, data curation, formal analysis. **Claudia Ernst:** methodology, investigation, data curation, formal analysis, writing – original draft. **Lisa Häfele:** investigation, data curation, formal analysis, writing – review and editing, software, visualization. **Lars Feuerbach:** conceptualization, methodology, data curation, formal analysis, writing – original draft, writing – review and editing. **Imke Grewe:** investigation. **Sumen Siqin:** conceptualization, data curation, formal analysis, writing – review and editing. **Karin Gunst:** investigation. **Gunjan Shukla:** conceptualization, writing – review and editing. **Veronika Frehtman:** investigation, data curation, formal analysis, writing – review and editing. **Claudia Tessmer:** investigation, data curation, formal analysis. **Florian Eichhorn:** data curation, formal analysis, resources. **Michael Allgäuer:** data curation, formal analysis, resources. **Alexander Brobeil:** data curation, formal analysis, resources. **Ilse Hofmann:** conceptualization, methodology, data curation, formal analysis, writing – review and editing, resources. **Barbara Leuchs:** conceptualization, methodology, data curation, formal analysis, writing – original draft, writing – review and editing. **Marc A. Schneider:** conceptualization, data curation, formal analysis, writing – original draft, writing – review and editing, resources, funding acquisition. **Timo Bund:** conceptualization, methodology, data curation, formal analysis, writing – original draft, writing – review and editing, funding acquisition, supervision, visualization, project administration.

## Funding

This work was supported by Dr. Rolf M. Schwiete Stiftung, grant 2022‐37; Hector Stiftung II, MED1804; World Cancer Research Fund International, SG_2021_059; ORYX Alpha GmbH & Co. KG; Deutsches Zentrum für Lungenforschung, 82DZL00402.

## Ethics Statement

The study was proved by the ethics committee of the University of Heidelberg (vote S‐270/2001 and vote S‐317/2023) and conducted in accordance with the Declaration of Helsinki (1975, revised in 2013) with written informed consent given by all participants.

## Conflicts of Interest

Michael Allgäuer received compensation for advisory board activity at AbbVie Inc. and speaker's honoraria from Boehringer Ingelheim AG & Co. K.G. The other authors declare no conflicts of interest.

## Supporting information


**Table S1:** Cohort descriptives. Description of clinicoepidemiological data for the paired tumor/peritumor cohort and control cohort (tissue adjacent to Hamartoma) used of immunohistological detection including smoking status.
**Table S2:** Patient/donor information. Sample information of the paired tumor/peritumor and control samples (tissue adjacent to Hamartoma) including sex, age, diagnosis and smoking status (PY = pack‐years).
**Table S3:** IHC DAB detection applied on clinical lung tissues. Peritumor ‘NG’ and tumor tissues “T” from lung cancer patients as well as tissues from non‐cancer individuals (tissues adjacent to hamartoma, “Hama”) were stained with eight Rep monoclonal antibodies (Rep AB 1, 2, 3, 7, 10, 11, 15 and 21) and anti‐CD68 antibody: “N/A”—missing FFPE tissues/sections, “✕”—no detection performed or detection results excluded due to poor tissue quality, “✓” indicates that the detection was performed (including information on clinical cohort collections).
**Table S4:** Detection of BMMF reads identified in PCAWG RNA‐seq and WGS data and association with BMMF templates. List of BMMF isolates covered by either BMMF1 (blue), BMMF2 (orange) or other BMMF‐ associated reads (white) detected in LUAD and LUSC tissues by D‐ViSioN in PCAWG RNA‐seq (A) and PCAWG WGS (B) data (reads mapping to more than one BMMF template counted separately).
**Table S5:** Detection of BMMF reads identified in scRNA‐seq data and association with BMMF templates. List of BMMF isolates covered by either BMMF1 (blue), BMMF2 (orange) or other BMMF‐ associated reads (white) detected in scRNA‐seq data from LUAD tissues and metastasis by D‐ViSioN pipeline (reads mapping to more than one BMMF template counted separately).
**Table S6:** List of genes used for differentiation of M1‐ or M2‐like macrophages. Genes used for differentiation of M1‐like (A) or M2‐like macrophages (B) (selection based on [28]).
**Figure S1:** Immunohistochemical detection of BMMF Rep in paired peritumor and tumor tissues of a lung cancer patient. Consecutive peritumor and tumor tissue sections (FFPE) of a representative lung cancer patient after DAB detection with anti‐Rep antibody AB 10 or isotype control.
**Figure S2:** Blocking of anti‐Rep antibody detection by pre‐incubation with Rep in DAB IHC. (A) Consecutive lung cancer peritumor tissue sections were immunohistologically stained with anti‐Rep AB 3 antibody after pre‐incubation with purified H1MSB.1 protein or incubation with the elution buffer, alone (urea buffer). For better orientation, identical tissue areas are marked with I–II and enlarged (3.5‐fold). (B) Quantification of the DAB positive area (%). Pre‐incubation with purified Rep (17 μg/mL) decreases the detection by around 55% (median of DAB‐stained area: 8.9 vs. 4.0) when compared to the control (Mann–Whitney *p* = 0.016).
**Figure S3:** Immunohistochemical detection of BMMF Rep and CD68 in peritumor tissues. Consecutive peritumor tissue sections (FFPE) from lung cancer patients were treated with anti‐Rep AB 3, anti‐CD68 or isotype control antibody or subjected to H&E staining. Tissues of 3 representative patients are shown. The detection pattern of CD68 generally overlaps with the detection pattern of Rep, but in some areas, CD68^+^ cells do not exhibit Rep expression (regions marked with red arrows) (for patients 12SI9R & NK1IKR same tissues areas as in Figure 1 were selected for better comparison of tissue markers).
**Figure S4:** Immunohistochemical detection of MARCO and CD11b in peritumoral lung tissue. FFPE sections of peritumoral tissues of two representative lung cancer patients stained by DAB immunohistochemistry using anti‐MARCO or anti‐CD11b antibodies, together with corresponding isotype controls. Scale bars, 50 μm.
**Figure S5:** Immunohistochemical detection of BMMF Rep in peritumor tissues. DAB detection of lung peritumor tissue sections (FFPE) of 2 representative patients with Rep AB 20 and 21 (raised against C1HB.4 Rep) and AB 22 and 23 (raised against H1MSB.2, C1MI.3M.1, C1MI.9M.1 Rep).
**Figure S6:** Co‐immunofluorescence detection of Rep, MARCO and CD11b in peritumoral lung tissues. (A) Tissue sections from two representative lung cancer patients (peritumoral regions) stained by immunofluorescence microscopy using anti‐Rep (AB3, red) in combination with either anti‐MARCO or anti‐CD11b antibodies (green), together with DAPI nuclear staining (blue). Merged images and enlarged views of selected regions (dashed boxes, two‐fold enlargement) are shown. Scale bars, 50 μm. (B) Quantification of Rep^+^ cells in the two individual tissue staining setups with MARCO or CD11b in consecutive peritumoral tissues (*n* = 10 representative tissues). The slight observable difference between Rep positive cell numbers between MARCO and CD11b quantification setups may be explained by the preference for interstitial tissue parts for the CD11b quantifications in the individual cuts. Correlation plots for MARCO^+^ (C) and CD11b^+^ (D) cells in peritumor tissues plotted against the number of Rep^+^ cells in individual patients (*n* = 10).
**Figure S7:** Quantification CD68^+^ CD163^‐^ (M1‐like) cells based on co‐immunofluorescence microscopy. Semi‐automated quantification of macrophages differentiated into CD68^+^CD163^‐^ M1‐like cells (left) and further subgrouping into Rep^+^ M1‐like macrophages (center) and Rep^‐^ M1‐like macrophages (right). One‐way ANOVA test used for statistical analysis. Significance levels: *p < 0.05, **p < 0.01, ***p < 0.001, ****p < 0.0001.
**Figure S8:** Immunohistochemical detection of BMMF Rep together with a marker of oxidative DNA damage. DAB anti‐Rep (brown) co‐detection together with a (surrogate) marker of oxidative DNA damage (8‐Hydroxyguanosine, 8OHdG, red) in two representative peritumor tissues of lung cancer patients (two loci, each). Areas with increased densities of Rep‐ and 8OHdG‐stained alveolar macrophages are marked with yellow asterisks, surrounding Rep‐negative but 8OHdG‐stained epithelial cells with red arrowheads and Rep^‐^/8OHdG^‐^ cells in the periphery with black arrowheads.
**Figure S9:** Rep expression in no smokers, ex‐smokers and smokers including differentiation by sex. Scatter plots of Rep^+^ cells (A), CD68^+^CD163^‐^ M1‐like cells (B), CD68^+^CD163^+^ M2‐like cells (C) and Rep^+^CD68^+^CD163^+^ cells (D) in no smokers (*n* = 7), ex‐smokers (*n* = 23) and smokers (*n* = 12). (E–H) Ex‐smokers and smokers were divided into low and high‐dose based on PY (pack‐years—a clinical measure of tobacco exposure, > 30 PY used as cut‐off for high smokers/ex‐smokers). (I–N) Scatter plots depicting Rep^+^ cells (I), CD68^+^ cells (J), CD68^+^CD163^‐^ M2‐like cells (K), CD68^+^CD163^‐^ M1‐like cells (L), Rep^+^CD68^+^CD163^+^ cells (M) and Rep^+^CD68^+^CD163^‐^ cells (N) in no smokers, ex‐smokers and smokers with further differentiation by sex. One‐way ANOVA used for the statistical analysis. No significant differences detected.
**Figure S10:** Correlation plots for the number of Rep^+^ (A), CD68^+^CD163^‐^ (B) and CD68^+^CD163^+^ (C) cells in peritumor tissues of actual smokers (*n* = 11) and of Rep^+^ (D), CD68^+^CD163^‐^ (E) and CD68^+^CD163^+^ (F) cells in peritumor tissues of ex‐smokers (*n* = 21) plotted against the intensity of smoking (pack‐years, PY) of the respective individual (statistical test by simple linear regression and Spearman's correlation).
**Figure S11:** Rep correlation with clinical‐epidemiological parameters. (A) Quantification of Rep^+^ cells in peritumor (9 females, 19 males) and control tissues (7 females, 12 males) grouped by sex (Mann–Whitney test: ns). Correlations of Rep^+^ cell numbers with age of female (*n* = 9, pink) and male (*n* = 19, blue) patients, together (B), or split for female (C) and male (D) patients (simple linear regression and Spearman correlation). (E) Stratification of Rep+ cell for NSCLC (*n* = 19) or SCLC (*n* = 7) patients.
**Figure S12:** Immunoblotting based on lysates from paired lung cancer tumor and peritumor tissues and tissues adjacent to Hamartoma. (A) Immunoblotting of frozen lung samples from Thorax Klinik project 3359 with Rep AB 10 and β‐actin. (B) Immunoblot of frozen lung samples from Thorax Klinik project 3359 with Rep AB 3 and β‐actin (left: same blots as in Figure 4R, for comparison). (C) Immunoblot of frozen lung samples from Thorax Klinik project 3694 with Rep AB 10 and β‐actin. Twenty cancer patients and 19 non‐cancer individuals tested, in total.
**Figure S13:** Detection of BMMF proteins by immunoblotting and mass spectrometry analysis. (A) WB results for Rep AB 3 for peritumor “P” and tumor “T” tissues from 3 lung cancer patients and 3 control tissues from non‐cancer individuals (adjacent to Hamartoma, “H”, same data as in Figure 4R, for comparison). Frozen tissues used for lysate preparation. (B) Purified H1MSB.1 Rep protein was spiked into lung tissue lysates before immunodetection. The band of positive control overlaps the lower band at ~37 kDa observed for the tissue lysates. Rep AB 3 used for detection. (C) Blocking of anti‐Rep antibody detection (4 μg/mL antibody AB 10) by co‐ incubation of WB membrane with purified H1MSB.1 Rep protein (17 ug/ml). (D) Detection of WB bands in one hamartoma and one peritumor tissue lysate after immunoprecipitation with Rep AB 10 or AB 2. (E) Mass spectroscopy‐detectable BMMF1 Rep peptide (LEEQFTQYDIEQISGLSSAYAVR) identified in the peritumor sample also after immunoprecipitation with anti‐Rep antibodies AB 2 and AB 10 (9 detection events).
**Figure S14:** Histologic detection of BMMF RNA by in situ hybridization in peritumor tissues of lung cancer patients. Peritumor lung tissues (FFPE) stained with RNA sense and anti‐sense probes directed against H1MSB.1 and H1MSB.2. Probes against dapB (4‐hydroxy‐tetrahydrodipicolinate reductase, negative) and PPIB (Peptidyl‐ prolyl cis‐trans isomerase B, positive) were used as controls.
**Figure S15:** Characteristics of BMMF reads identified in macrophages and T‐cells of lung cancer patients by scRNA‐seq. Donut plots illustrating numbers of patients and cells harboring BMMF reads grouped into macrophages (A) and T‐cells (B) prepared from biopsies of primary lung tumor and metastasis (C = cells, *P* = patients).

## Data Availability

The raw mass spectrometry data generated in this study have been deposited in the PRoteomics IDEntifications Database (PRIDE, EMBL‐EBI) database under accession number PXD077096 [58]. R code is publicly available on GitHub (https://github.com/nivescecere/M1‐M2‐BMMF‐Lung.git). Data sources and handling of the publicly available datasets used in this study are described in the Materials and Methods. Further details and other data that support the findings of this study are available from the corresponding authors upon request.
