# Supplementary material for: Lung Cancer Patients Display Increased Expression of Bovine Meat and Milk Factor (BMMF) Proteins in Peritumor Alveolar Macrophages
Source: Int J Cancer. 2026 Jun 1;159(8):2036–53. doi: 10.1002/ijc.70574 (PMC13495879; doi:10.1002/ijc.70574)
Supplement: Supplementary file 1 [file IJC-159-2036-s001.pdf]

Lung cancer patients display increased expression of Bovine Meat and Milk Factor (BMMF) proteins in peritumor alveolar macrophages

Nives Cecere, Kristina Alikhanyan, Claudia Ernst, Lisa Häfele, Lars Feuerbach, Imke Grewe, Sumen Siqin, Karin Gunst, Gunjan Shukla, Veronika Frehtman, Claudia Tessmer, Florian Eichhorn, Michael Allgäuer, Alexander Brobeil, Ilse Hofmann, Barbara Leuchs, Marc A. Schneider, Timo Bund

Supporting information

Tables S1 – S6

Figures S1 – S15

Table S1

Table S1. Cohort descriptives. Description of clinicoepidemiological data for the paired tumor/peritumor cohort and control cohort (tissue adjacent to Hamartoma) used of immunohistological detection including smoking status.

		Paired tumor/Peritumor		Control (Hamartoma)		All	
Variable/Level		n	%	n	%	n	%
Sex	Male	22	59.5	12	63.2	34	60.7
	Female	14	37.8	7	36.8	21	37.5
	Not available	1	2.7	0	0.0	1	1.8
	All	37	100.0	19	100.0	56	100.0
Age	40-55	7	18.9	7	36.8	14	25.0
	56-65	13	35.1	7	36.8	20	35.7
	66-79	14	37.8	5	26.3	19	33.9
	80-89	2	5.4	0	0.0	2	3.6
	Not available	1	2.7	0	0.0	1	1.8
	All	37	100.0	19	100.0	56	100.0
Diagnosis	Adenocarcinoma	11	29.7				
	Adenocarcinoma with neuroendcr. Diff.	1	2.7				
	Large cell lung carcinoma	1	2.7				
	Large Cell Neuroenocr. Lung Carc. (LCNEC)	2	5.4				
	Small cell lung cancer (SCLC)	6	16.2				
	Non-small cell lung carc., AD (NSCLC)	5	13.5				
	Non-small cell lung carc,, SCC (NSCLC)	5	13.5				
	Squamous cell carcinoma (SCC)	5	13.5				
	Not available	1	2.7				
	All	37	100.0				
Smoking status	Non Smoker	4	16.7	3	17.6	7.0	17.1
	Smokers	7	29.2	4	23.5	11.0	26.8
	Ex-Smoker	13	54.2	10	58.8	23.0	56.1
	All	24	100.0	17	100.0	41	100.0
Packages per year (PY)	0-20	9	37.5	9	52.9	18.0	43.9
	21-35	3	12.5	3	17.6	6.0	14.6
	36-60	5	20.8	4	23.5	9.0	22.0
	61-90	5	20.8	1	5.9	6.0	14.6
	Not available	2	8.3				
	All	24	100.0	17	100.0	41	100.0

Table S2

Table S2. Patient/donor information. Sample information of the paired tumor/peritumor and control samples (tissue adjacent to Hamartoma) including sex, age, diagnosis and smoking status (PY= pack-years).

Sample ID	Sex	Age	Diagnosis	Smoking Status	PY
10X1PE	m	48	Hamartoma	Ex-Smoker	30
11F3TZ	w	68	Squamous cell carcinoma (SCC)	Ex-Smoker	60
11F3TZ	w	68	Squamous cell carcinoma (SCC)	Ex-Smoker	60
123UWA	m	59	Hamartoma	Ex-Smoker	5
12SI9R	w	56	Large cell lung carcinoma	Smoker	40
13DRL6	w	47	Hamartoma	Ex-Smoker	5
14CIFM	w	49	Non-small cell lung carcinoma, adenocarcinoma (NSCLC)	Ex-Smoker	50
14ZOKY	w	74	Adenocarcinoma	Non Smoker	
14ZOKY	w	74	Adenocarcinoma	Non Smoker	
16PS7Y	w	51	Non-small cell lung carcinoma, adenocarcinoma (NSCLC)	not specified	
1A3RXW	w	57	Hamartoma	Ex-Smoker	30
1AESOW	m	60	Squamous cell carcinoma (SCC)	Ex-Smoker	50
1AESOW	m	60	Squamous cell carcinoma (SCC)	Ex-Smoker	50
1CVVKA	w	67	Hamartoma	not specified	
1DVRTK	m	71	Non-small cell lung carcinoma, squamous cell carcinoma (NSCLC)	Ex-Smoker	10
1FAVEM	m	65	Non-small cell lung carcinoma, squamous cell carcinoma (NSCLC)	not specified	
1G944Y	m	55	Hamartoma	Smoker	80
1IK3RV	m	77	Non-small cell lung carcinoma, adenocarcinoma (NSCLC)	Non Smoker	
1IX6KF	w	78	Non-small cell lung carcinoma, adenocarcinoma (NSCLC)	Non Smoker	
1LYLZF	m	63	Adenocarcinoma	Smoker	80
1LYLZF	m	63	Non-small cell lung carcinoma, adenocarcinoma (NSCLC)	Ex-Smoker	80
1NZV3L	w	64	Adenocarcinoma with neuroendocrine differentiation	Ex-Smoker	
1OE7G1	w	78	Hamartoma	Ex-Smoker	5
1PZHGD	m	69	Small cell lung cancer (SCLC)	Ex-Smoker	70
1RGUL5	w	71	Hamartoma	Non Smoker	
1SRX8R	m	63	Small cell lung cancer (SCLC)	Ex-Smoker	25
1TA5ZI	w	76	Squamous cell carcinoma (SCC)	not specified	
1TA5ZI	w	76	Squamous cell carcinoma (SCC)	not specified	
1W2LHA	m	62	Adenocarcinoma	Smoker	
1W2LHA	m	62	Adenocarcinoma	Smoker	
1XFDVM	m	48	Hamartoma	not specified	
2V6GGQ	m	45	Large Cell Neuroendocrine Lung Carcinoma (LCNEC)	Smoker	10
3N4U9G	m	68	Hamartoma	not specified	
4B8HD0	m	74	Adenocarcinoma	Smoker	15
4B8HD0	m	74	Adenocarcinoma	Smoker	15
4E3TIC	w	76	Hamartoma	Ex-Smoker	20
57D3UO	m	63	Adenocarcinoma	Ex-Smoker	34
57D3UO	m	63	Adenocarcinoma	Ex-Smoker	34
6LBF64	m	63	Small cell lung cancer (SCLC)	Ex-Smoker	40
9DFQ5K	m	55	Hamartoma	Smoker	45
9SSVPV	m	59	Hamartoma	Ex-Smoker	40
B3X2KD	m	60	Adenocarcinoma	Non Smoker	
B3X2KD	m	60	Adenocarcinoma	Non Smoker	
E6B6PL	m	64	Adenocarcinoma	Ex-Smoker	20
E6B6PL	m	64	Adenocarcinoma	Ex-Smoker	20
HC87BC	m	50	Adenocarcinoma	Ex-Smoker	15
HC87BC	m	50	Adenocarcinoma	Ex-Smoker	15
HHU68Y	w	64	Squamous cell carcinoma (SCC)	Ex-Smoker	90
HHU68Y	w	64	Non-small cell lung carcinoma, squamous cell carcinoma (NSCLC)	Ex-Smoker	90
IUDT9Z	m	60	Hamartoma	Smoker	40
J0ZIYM	m	56	Hamartoma	Smoker	15
JGS1F3	m	72	Squamous cell carcinoma (SCC)	Ex-Smoker	20
JGS1F3	m	72	Squamous cell carcinoma (SCC)	Ex-Smoker	20
MO57SD	m	78	Non-small cell lung carcinoma, squamous cell carcinoma (NSCLC)	Ex-Smoker	45
NC2CFC	m	60	Hamartoma	Ex-Smoker	30
NK1IKR	m	64	Adenocarcinoma	Smoker	45
NK1IKR	m	65	Non-small cell lung carcinoma, adenocarcinoma (NSCLC)	Smoker	45
NKPU2X	w	80	Adenocarcinoma	not specified	
NKPU2X	w	80	Adenocarcinoma	not specified	
O6QVVN	m	68	Large Cell Neuroendocrine Lung Carcinoma (LCNEC)	Ex-Smoker	

ORJWVD	w	52	Small cell lung cancer (SCLC)	Smoker	60
OVHCKC	m	70	Small cell lung cancer (SCLC)	Ex-Smoker	30
PJ841U	m	51	Hamartoma	Ex-Smoker	45
RGN43J	m	54	Squamous cell carcinoma (SCC)	Ex-Smoker	30
RGN43J	m	54	Squamous cell carcinoma (SCC)	Ex-Smoker	30
TJHWQ0	w	75	Adenocarcinoma	Non Smoker	
TJHWQ0	w	75	Adenocarcinoma	Non Smoker	
TXZBEE	m	44	Hamartoma	Ex-Smoker	10
UJEQ8N	m	62	Squamous cell carcinoma (SCC)	Smoker	
UJEQ8N	m	61	Non-small cell lung carcinoma, squamous cell carcinoma (NSCLC)	Smoker	80
WPPSS6	w	61	Hamartoma	not specified	
XNB56E	w	50	Small cell lung cancer (SCLC)	Smoker	70
XNB56E	w	50	Small cell lung cancer (SCLC)	Smoker	70
ZCZ8WH	m	69	Non-small cell lung carcinoma, squamous cell carcinoma (NSCLC)	Non Smoker	

Table S3

Table S3. IHC DAB detection applied on clinical lung tissues. Peritumor ‘NG’ and tumor tissues ‘T’ from lung cancer patients as well as tissues from non-cancer individuals (tissues adjacent to hamartoma, ‘Hama’) were stained with eight Rep monoclonal antibodies (Rep AB 1, 2, 3, 7, 10, 11, 15 and 21) and anti-CD68 antibody: ‘N/A’ - missing FFPE tissues/sections, ‘X’ – no detection performed or detection results excluded due to poor tissue quality, ‘✓′ indicates that the detection was performed (including information on clinical cohort collections).

Patient	Tissue	AB 3	AB 10	AB 1	AB 7	AB 11	AB 15	AB 2	AB 21	CD68
1IX6KF	T	✓	✓	✓	✓	✓	✓	✓	✓	✓
	NG	✓	✓	✓	✓	✓	X	✓	✓	✓
16PS7Y	T	✓	✓	✓	✓	✓	✓	✓	✓	✓
	NG	N/A	N/A	N/A	N/A	N/A	N/A	N/A	N/A	N/A
1LYLZF	T	✓	✓	✓	✓	✓	✓	✓	✓	✓
	NG	✓	✓	✓	✓	✓	X	✓	✓	✓
1FAVEM	T	✓	✓	✓	✓	✓	✓	✓	✓	✓
	NG	✓	✓	✓	✓	✓	X	✓	✓	✓
1DVRTK	T	✓	✓	✓	✓	✓	✓	✓	✓	✓
	NG	✓	✓	✓	✓	✓	X	✓	✓	✓
ORJWVD	T	✓	✓	✓	✓	✓	✓	✓	✓	✓
	NG	✓	✓	✓	✓	✓	X	✓	✓	✓
1PZHGD	T	✓	✓	✓	✓	✓	✓	✓	✓	✓
	NG	✓	✓	✓	✓	✓	X	✓	✓	✓
14CIFM	T	✓	✓	✓	✓	✓	X	X	X	✓
	NG	N/A	N/A	N/A	N/A	N/A	N/A	N/A	N/A	N/A
UJEQ8N	T	✓	✓	✓	✓	✓	✓	✓	✓	✓
	NG	✓	✓	✓	✓	✓	X	✓	✓	✓
OVHCKC	T	✓	✓	✓	✓	✓	✓	✓	✓	✓
	NG	✓	✓	✓	✓	✓	X	✓	✓	✓
1IK3RV	T	✓	✓	✓	✓	✓	✓	✓	✓	✓
	NG	✓	✓	✓	✓	✓	X	✓	✓	✓
HHU68Y	T	✓	✓	✓	✓	✓	✓	✓	✓	✓
	NG	✓	✓	✓	✓	✓	X	✓	✓	✓
NK1IKR	T	✓	✓	✓	✓	✓	✓	✓	✓	✓
	NG	✓	✓	✓	✓	✓	X	✓	✓	✓
6LBF64	T	✓	✓	✓	✓	✓	✓	✓	✓	✓
	NG	✓	✓	✓	✓	✓	X	✓	✓	✓
XNB56E	T	✓	✓	✓	✓	✓	✓	✓	✓	✓
	NG	✓	✓	✓	✓	✓	X	✓	✓	✓
1SRX8R	T	✓	✓	✓	✓	✓	✓	✓	✓	✓
	NG	✓	✓	✓	✓	✓	X	✓	✓	✓
ZCZ8WH	T	✓	✓	✓	✓	✓	✓	✓	✓	✓
	NG	✓	✓	✓	✓	✓	X	✓	✓	✓
MO57SD	T	✓	✓	✓	✓	✓	✓	✓	✓	✓
	NG	✓	✓	✓	✓	✓	X	✓	✓	✓
O6QVVN	T	✓	✓	✓	✓	✓	X	✓	✓	✓
	NG	✓	✓	✓	✓	✓	X	X	X	✓
1NZV3L	T	✓	✓	✓	✓	✓	✓	✓	✓	✓
	NG	✓	✓	✓	✓	✓	X	✓	✓	✓
2V6GGQ	T	✓	✓	✓	✓	✓	✓	✓	✓	✓
	NG	✓	✓	✓	✓	✓	X	✓	✓	✓
12SI9R	T	✓	✓	✓	✓	✓	✓	✓	✓	✓
	NG	✓	✓	✓	✓	✓	X	✓	✓	✓
DTN9K4	T	✓	✓	✓	✓	✓	X	X	✓	✓
	NG	✓	✓	✓	✓	✓	X	✓	✓	✓

Patient	Tissue	AB 3	AB 10	AB 1	AB 7	AB 11	AB 15	AB 2	AB 21	CD 68
11F3TZ	T	✓	✓	✓	✓	✓	X	✓	✓	✓
	NG	✓	✓	✓	✓	✓	X	✓	✓	✓
1GCLAF	T	✓	✓	✓	✓	✓	X	✓	✓	✓
	NG	✓	✓	✓	✓	✓	X	✓	✓	✓
RGN43J	T	✓	✓	✓	✓	✓	X	✓	✓	✓
	NG	✓	✓	✓	✓	✓	X	✓	✓	✓
1TA5ZI	T	✓	✓	✓	✓	✓	X	✓	✓	✓
	NG	✓	✓	✓	✓	✓	X	✓	✓	✓
JGS1F3	T	✓	✓	✓	✓	✓	X	✓	✓	✓
	NG	✓	X	✓	✓	X	X	✓	✓	✓
57D3UO	T	✓	✓	✓	✓	✓	X	✓	✓	✓
	NG	N/A	N/A	N/A	N/A	N/A	N/A	N/A	N/A	N/A
1W2LHA	T	✓	✓	✓	✓	✓	X	✓	✓	✓
	NG	✓	✓	✓	✓	✓	X	✓	✓	✓
B3X2KD	T	✓	✓	✓	✓	✓	X	✓	✓	✓
	NG	✓	✓	✓	✓	✓	X	✓	✓	✓
TJHWQ0	T	✓	✓	✓	✓	✓	X	✓	✓	✓
	NG	✓	X	✓	X	✓	X	✓	✓	✓
14ZOKY	T	✓	✓	✓	✓	✓	X	✓	✓	✓
	NG	✓	✓	✓	✓	✓	X	✓	✓	✓
4B8HD0	T	X	X	X	X	X	X	✓	✓	✓
	NG	✓	✓	✓	✓	✓	X	✓	✓	✓
E6B6PL	T	✓	✓	✓	✓	X	X	✓	✓	✓
	NG	✓	✓	X	X	✓	X	✓	X	✓
NKPU2X	T	✓	✓	✓	✓	✓	X	✓	✓	✓
	NG	✓	X	✓	✓	X	X	✓	✓	✓
HC87BC	T	✓	✓	✓	✓	✓	X	✓	✓	✓
	NG	✓	X	✓	✓	X	X	✓	✓	✓
1RGUL5	Hama.	✓	✓	✓	✓	✓	X	✓	✓	✓
IUDT9Z	Hama.	✓	✓	✓	✓	✓	X	✓	✓	✓
4E3TIC	Hama.	✓	✓	✓	✓	✓	X	✓	✓	✓
1A3RXW	Hama.	✓	✓	✓	✓	✓	X	✓	✓	✓
1XFDVM	Hama.	✓	✓	✓	✓	✓	X	✓	✓	✓
TXZBEE	Hama.	✓	✓	✓	✓	✓	X	✓	✓	✓
NC2CFC	Hama.	✓	✓	✓	✓	✓	X	✓	✓	✓
10X1PE	Hama.	✓	X	✓	X	✓	X	✓	✓	✓
1G944Y	Hama.	✓	✓	✓	✓	✓	X	✓	✓	✓
J0ZIYM	Hama.	✓	✓	✓	✓	✓	X	✓	✓	✓
9SSVPV	Hama.	✓	✓	✓	✓	✓	X	X	✓	✓
3N4U9G	Hama.	✓	✓	✓	✓	✓	X	X	✓	✓
1OE7G1	Hama.	✓	✓	✓	✓	✓	X	X	✓	✓
PJ841U	Hama.	✓	✓	✓	✓	✓	X	X	✓	✓

Patient	Tissue	AB 3	AB 10	AB 1	AB 7	AB 11	AB 15	AB 2	AB 21	CD 68
9DFQ5K	Hama.	✓	✓	✓	✓	✓	X	✓	✓	✓
13DRL6	Hama.	✓	✓	✓	✓	✓	X	✓	✓	✓
WPPSS6	Hama.	✓	✓	✓	✓	✓	X	✓	✓	✓
123UWA	Hama.	✓	✓	✓	✓	✓	X	✓	✓	✓
1CVVKA	Hama.	✓	✓	✓	✓	✓	X	✓	✓	✓
15183	PT	✓	✓	✓	✓	✓	✓	✓	✓	✓
15184	PT	✓	✓	✓	✓	✓	✓	✓	✓	✓
15188	PT	✓	✓	✓	✓	✓	✓	✓	✓	✓
15192	PT	✓	✓	✓	✓	✓	✓	✓	✓	✓
15193	PT	✓	✓	✓	✓	✓	✓	✓	✓	✓
15194	PT	✓	✓	✓	✓	✓	✓	✓	✓	✓
15195	PT	✓	✓	✓	✓	✓	✓	✓	✓	✓
15196	PT	✓	✓	✓	✓	✓	✓	✓	✓	✓

NCT – Thorax Klinik Project 2750
NCT – Thorax Klinik Project 3359
NCT – Thorax Klinik Project 3694
NCT – Thorax Klinik Fresh Tissues

Table S4

Table S4. Detection of BMMF reads identified in PCAWG RNA-seq and WGS data and association with BMMF templates. List of BMMF isolates covered by either BMMF1 (blue), BMMF2 (orange) or other BMMF-associated reads (white) detected in LUAD and LUSC tissues by D-ViSioN in PCAWG RNA-seq ***(A)*** and PCAWG WGS ***(B)*** data (reads mapping to more than one BMMF template counted separately).

A

LUAD – RNA-seq	
Template	Hits
C1HB.4	2
MSSI1.162	1
C2MI.16B.10	1
pRGRH0636	1
Total	5

LUSC – RNA-seq	
Template	Hits
C1HB.4	9
C1MI.15M.1	1
pHD4	1
C1MI.3M.1	1
Total	12

B

LUAD – WGS

Primary tumor (n=4)	
Template	Hits
C1MI.4	2
C1MI.5.1	2
C1MI.2	1
C2MI.3A.1	1
C2MI.8A.1	1
pA85	1
H1MSB.1	1
Total	9

Normal tissues (total n=5)	
Template	Hits
C2MI.15B.16	2
C1HB.6.1	1
C1MI.5.1	1
C1MI.9M.1	1
C2MI.15B.17	1
C2MI.8B.4	1
H1MSB.1	1
H1MSB.2	1
Total	9

Normal tissues, blood derived (n=3)	
Template	Hits
C1HB.6.1	1
C1MI.5.1	1
H1MSB.1	1
C1MI.9M.1	1
H1MSB.2	1
Total	5

Normal tissues, solid tissues (n=2)	
Template	Hits
C2MI.15B.16	2
C2MI.15B.17	1
C2MI.8B.4	1
Total	4

LUSC – WGS

Primary tumor (n=20)	
Template	Hits
C1MI.3	25
Sphinx2.36	24
C1MI.9M.1	14
C1MI.2	12
H1MSB.2	12
C1MI.3M.1	11
pHD4	10
C1MI.15M.1	9
H1MSB.1	8
C1MI.5.1	5
C1MI.4	5
C1MI.9M.2	5
C1HB.6.1	4
C1HB.3	3
C1HB.4	3
C2MI.15B.1	2
C1HB.5	1
C2HB2	1
C2MI.15B.2	1
C2MI.4A.1	1
Total	156

Normal tissues (total n=15)	
Template	Hits
C1MI.3	11
C1MI.2	5
C1MI.3M.1	5
H1MSB.2	4
H1MSB.1	3
C1HB.3	3
Sphinx2.36	3
pHD4	3
C1MI.5.1	3
MSSI1.162	2
C1MI.4	2
C1MI.9M.1	2
C1HB.6.1	1
C1MI.9M.2	1
C2MI.7B.17	1
Total	49

Normal tissues, blood derived (n=7)	
Template	Hits
C1MI.3	6
C1MI.2	4
Sphinx2.36	3
pHD4	3
H1MSB.2	2
H1MSB.1	2
C1MI.4	2
Total	22

Normal tissues, solid tissues (n=8)	
Template	Hits
C1MI.3	5
C1MI.3M.1	5
C1HB.3	3
C1MI.5.1	3
H1MSB.2	2
MSSI1.162	2
C1MI.9M.1	2
C1HB.6.1	1
C1MI.9M.2	1
C2MI.7B.17	1
H1MSB.1	1
C1MI.2	1
Total	27

Table S5

Table S5. Detection of BMMF reads identified in in scRNA-seq data and association with BMMF templates. List of BMMF isolates covered by either BMMF1 (blue), BMMF2 (orange) or other BMMF-associated reads (white) detected in scRNA-seq data from LUAD tissues and metastasis by D-ViSioN pipeline (reads mapping to more than one BMMF template counted separately).

Template	Hits
MSSI1.162	1044
C1HB.4	157
C2MI.15B.16	83
C1MI.3M.1	72
C2MI.8A.1	62
HCBI9.212	43
C2MI.15B.10	39
C1MI.2	35
C1HB.3	29
H2HB7	24
C1MI.15M.1	23
C1MI.4	22
Sphinx2.36	21
C1MI.5.2	19
H1MSB.1	16
pHD4	15
H1MSB.2	14
C2MI.5B.8	11
MSSI2.225	10
C1MI.3	7
C2MI.1A.1	6
C2MI.9A.1	6
C2MI.16B.12	5
C2MI.15B.17	5

BMMF-Template	Hits
C2MI.15B.14	5
C2MI.9B.14	5
C2MI.13B.1	4
C2MI.4A.1	4
C2MI.9B.4	4
pRGRH0103	3
C2MI.10A.1	3
C2MI.9B.7	3
C2MI.5A.2	3
C2HB2	3
C1HB.5	3
C2MI.10A.2	2
C2MI.15B.12	2
C2MI.15B.4	2
C2MI.15B.3	2
C2MI.5A.4	2
C1HB.6.1	2
C2MI.3A.1	1
C2MI.1A.3	1
C2MI.1A.2	1
C2MI.16B.10	1
C2MI.15B.2	1
C1MI.15M.2	1
**Total**	**1826**

Table S6

Table S6. List of genes used for differentiation of M1- or M2-like macrophages. Genes used for differentiation of M1-like ***(A)*** or M2-like macrophages ***(B)*** (selection based on [28])

A

CCR7	PLA1A	TRAIL	CSPG2	IL12B	CCL5
IL2RA	OASL	IL6	APOL3	IL15RA	CCL15
IL15RA	CHI3L2	CCL20	IGFBP4	SLC31A2	PSMB9
IL7R	HSD11B1	PBEF1	APOL1	OAS2	PSMA2
CXCL11	AK3	ECGF1	PDGFA	PTX3	HSXIAPAF1
CCL19	SPHK1	BCL2A1	EDN1	INDO	SLC7A5
CXCL10	PFKFB3	FAS	APOL2	SLC21A15	HESX1
CXCL9	PSME2	BIRC3	INHBA	SLC2A6	IRF1
TNF	PFKP	GADD45G	APOL6	ATF3	IRF7

B

GPR86	P2RY14	SLC21A9	CERK	FN1	DCL-1
P2RY5	DCSIGN	SLC4A7	HS3ST2	FGL2	MSR1
TGFBR2	CLECSF13	SLC38A6	LTA4H	GAS7	CXCR4
HRH1	MS4A4A	CTSC	CA2	EGR2	DECTIN1
TLR5	MRC1	HEXB	ALOX15	MAF	LIPA
HS3ST1	TGFBI	SEPP1	CHN2	IGF1	ADK
CCL13	CCL18	TPST2	HNMT	CCL23	

Figure S1

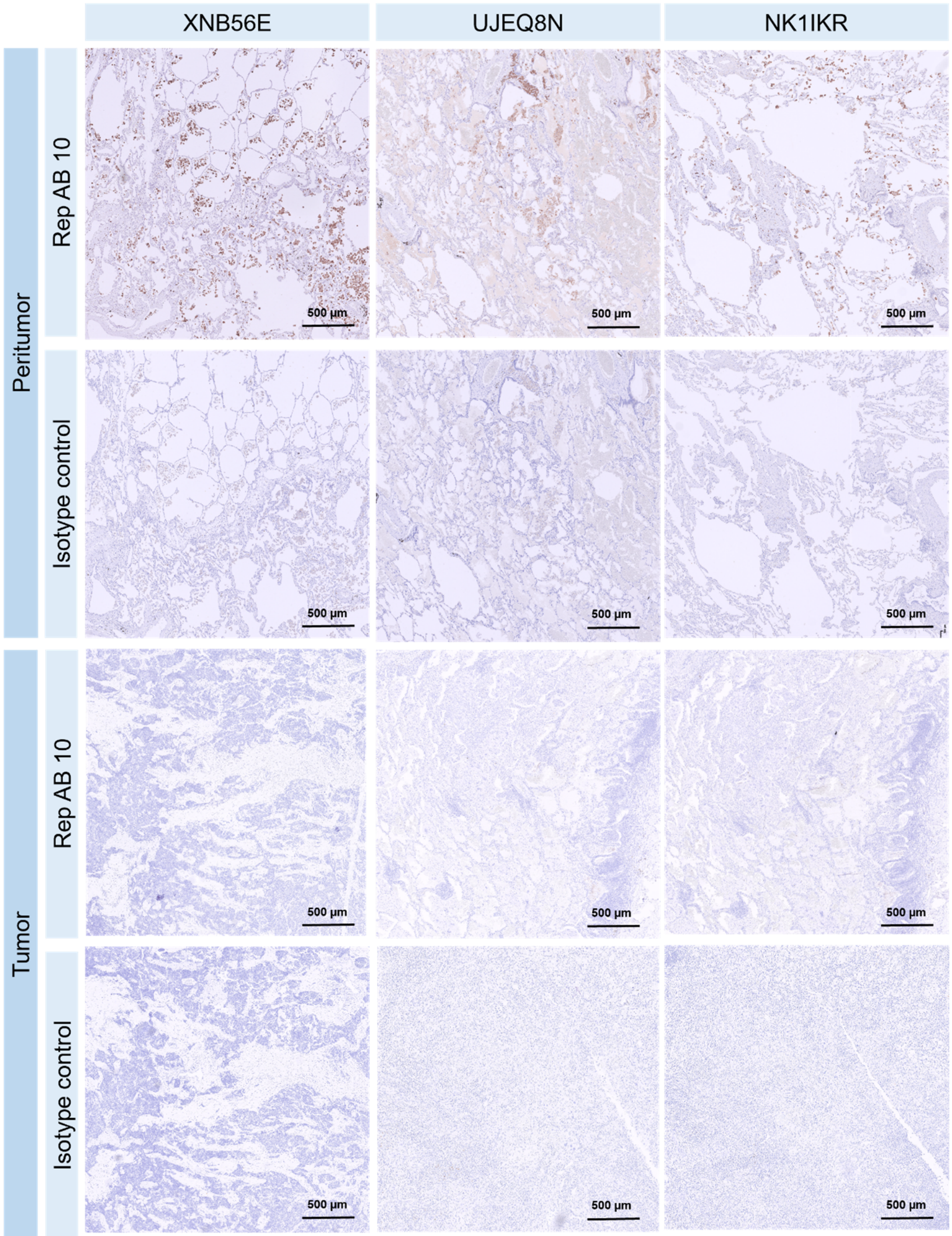

Figure S1. Immunohistochemical detection of BMMF Rep in paired peritumor and tumor tissues of a lung cancer patient. Consecutive peritumor and tumor tissue sections (FFPE) of a representative lung cancer patient after DAB detection with anti-Rep antibody AB 10 or isotype control.

Figure S2

A

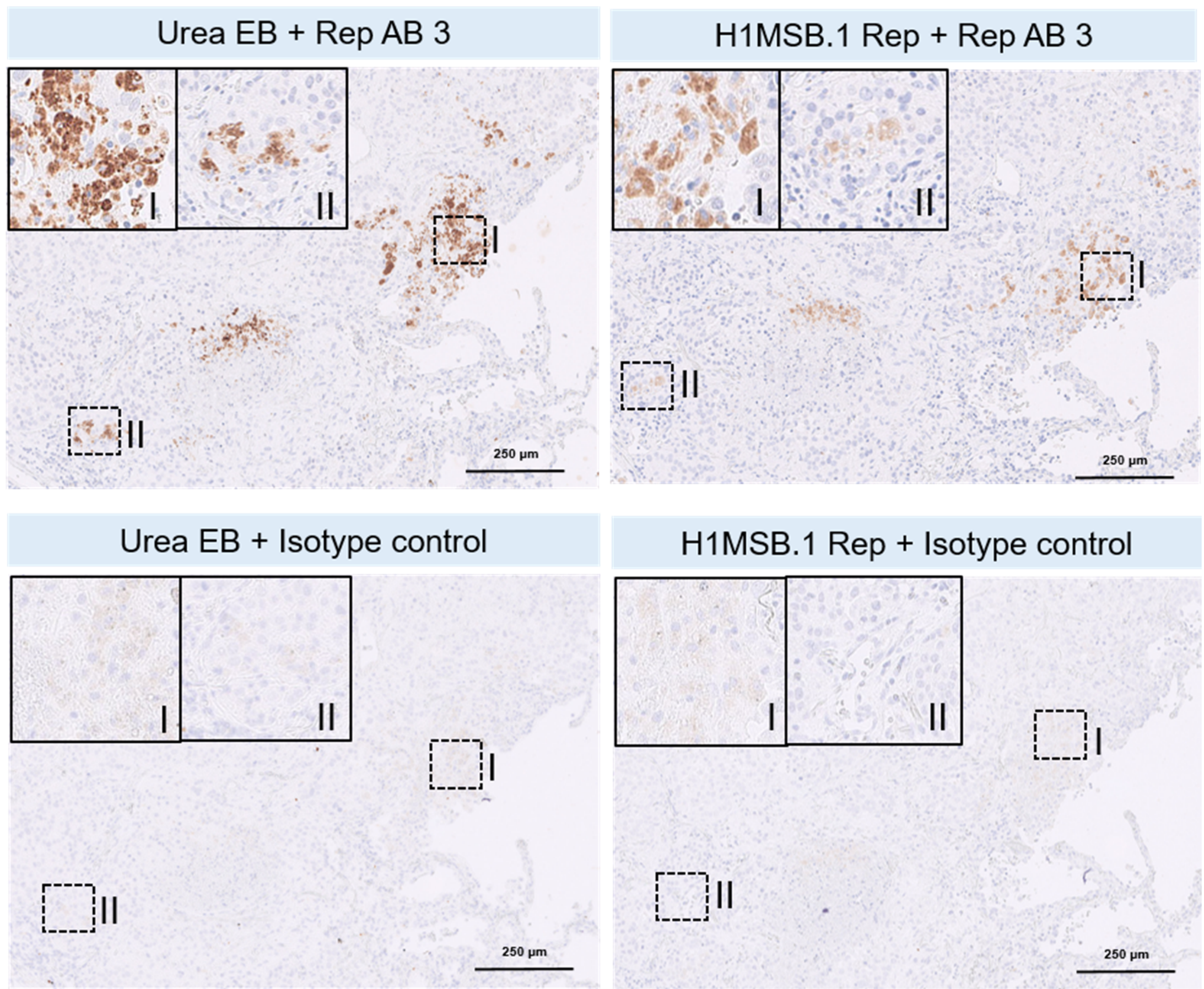

B

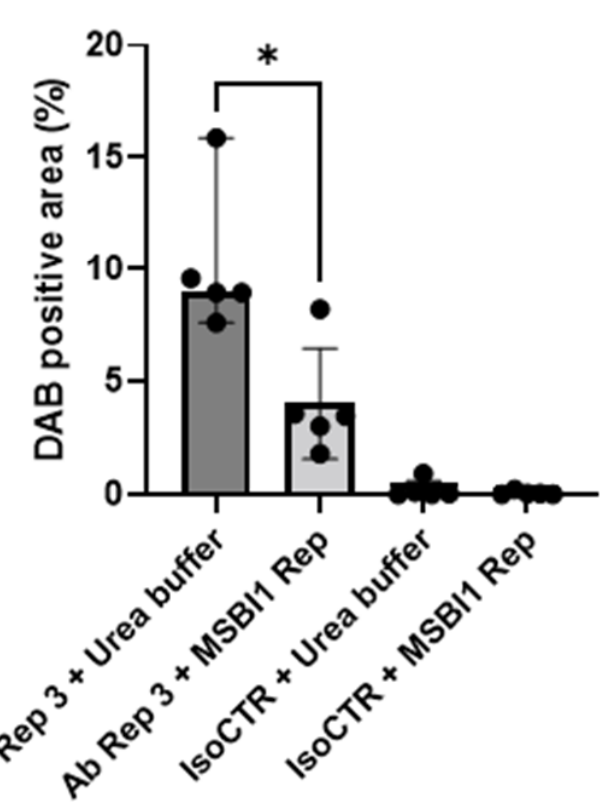

Figure S2. Blocking of anti-Rep antibody detection by pre-incubation with Rep in DAB IHC. ***(A)*** Consecutive lung cancer peritumor tissue sections were immunohistologically stained with anti-Rep AB 3 antibody after pre-incubation with purified H1MSB.1 protein or incubation with the elution buffer, alone (urea buffer). For better orientation, identical tissue areas are marked with I-II and enlarged (3.5-fold). ***(B)*** Quantification of the DAB positive area (%). Pre-incubation with purified Rep (17 µg/ml) decreases the detection by around 55% (median of DAB-stained area: 8.9 vs. 4.0) when compared to the control (Mann-Whitney P=0.016).

Figure S3

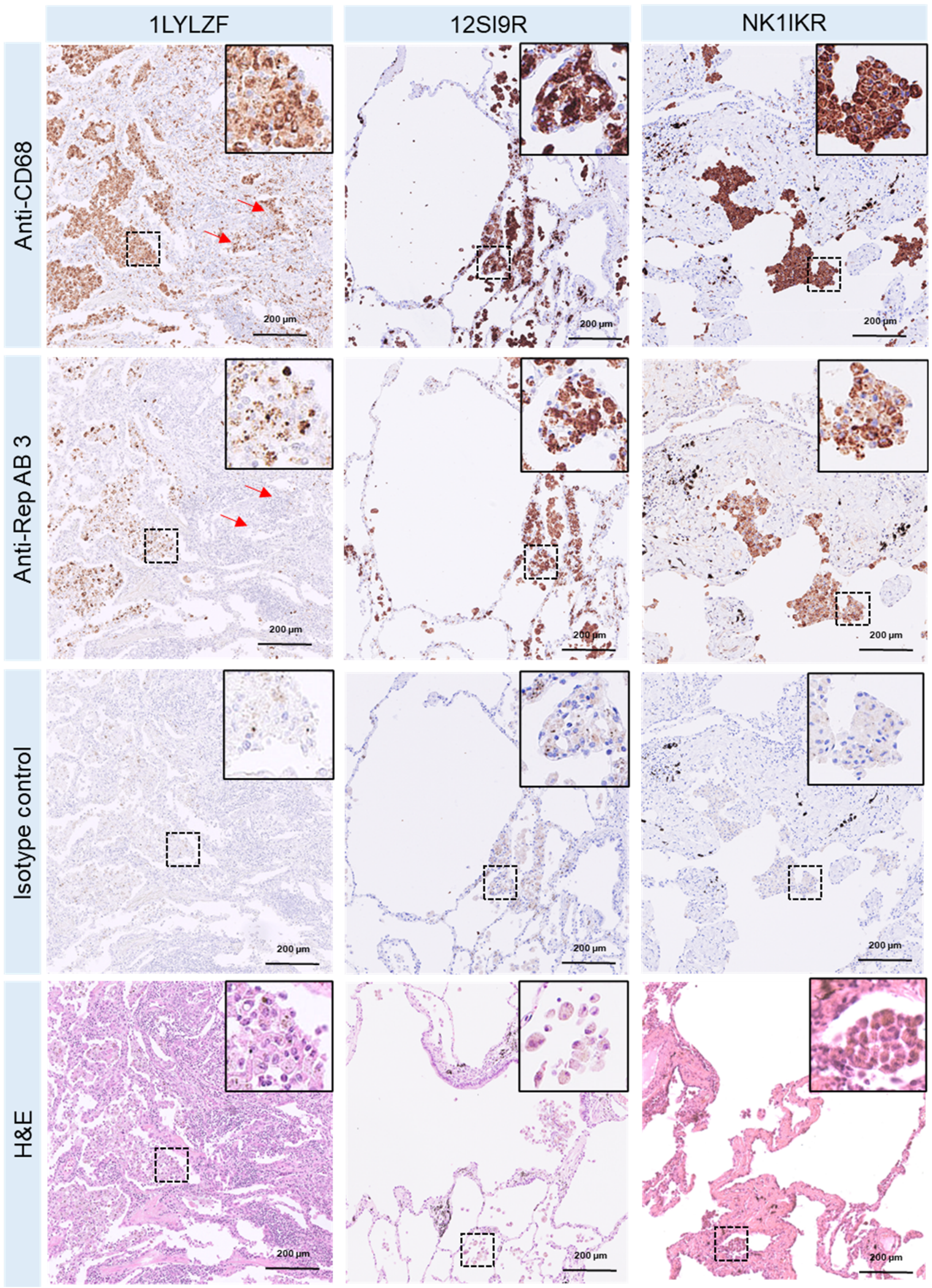

Figure S3. Immunohistochemical detection of BMMF Rep and CD68 in peritumor tissues. Consecutive peritumor tissue sections (FFPE) from lung cancer patients were treated with anti-Rep AB 3, anti-CD68 or isotype control antibody or subjected to H&E staining. Tissues of 3 representative patients are shown. The detection pattern of CD68 generally overlaps with the detection pattern of Rep, but in some areas, $CD68^+$ cells do not exhibit Rep expression (regions marked with red arrows) (for patients 12SI9R & NK1IKR same tissues areas as in Fig. 1 were selected for better comparison of tissue markers).

Figure S4

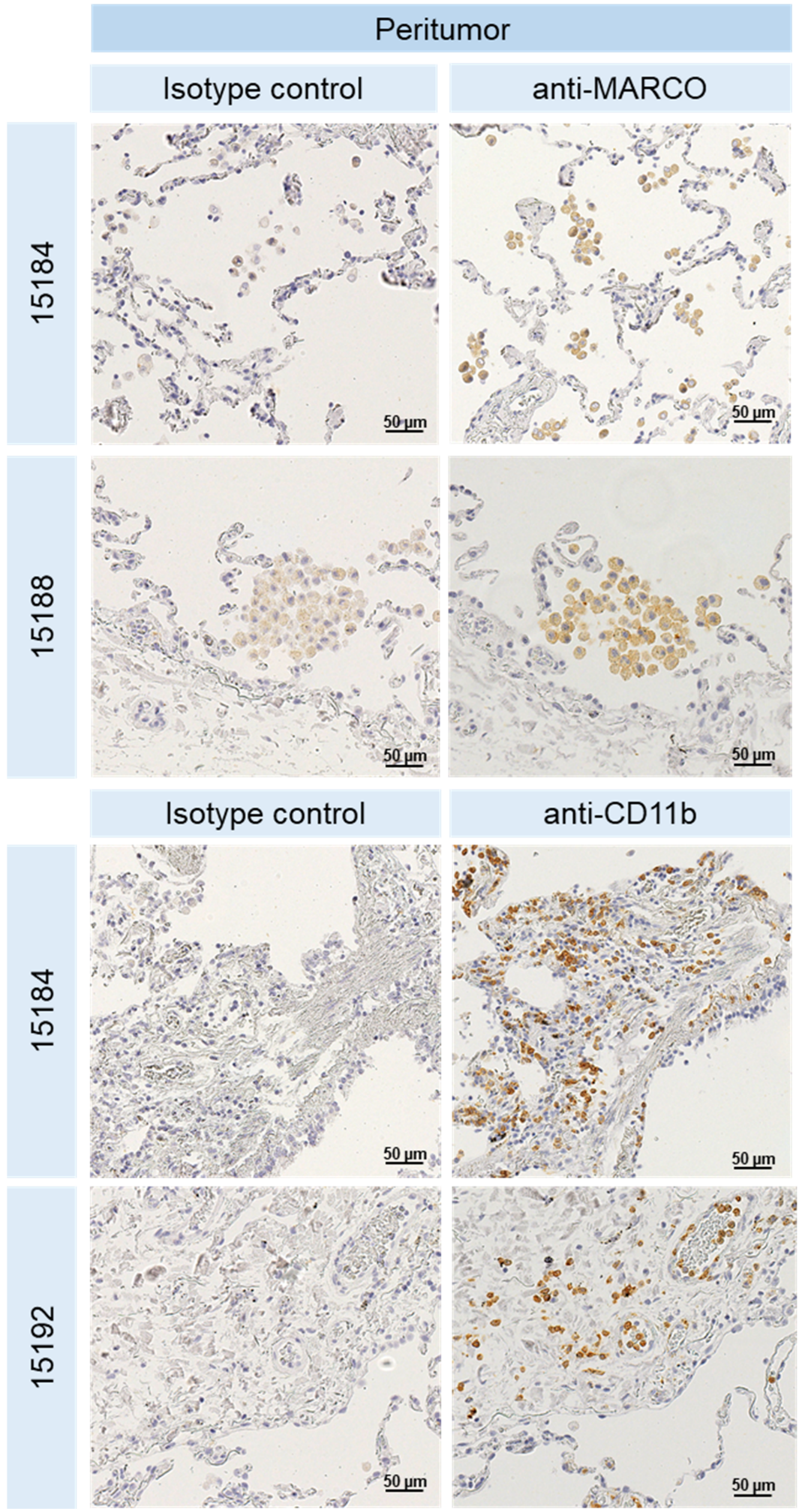

Figure S4. Immunohistochemical detection of MARCO and CD11b in peritumoral lung tissue. FFPE sections of peritumoral tissues of two representative lung cancer patients stained by DAB immunohistochemistry using anti-MARCO or anti-CD11b antibodies, together with corresponding isotype controls. Scale bars, 50 µm.

Figure S5

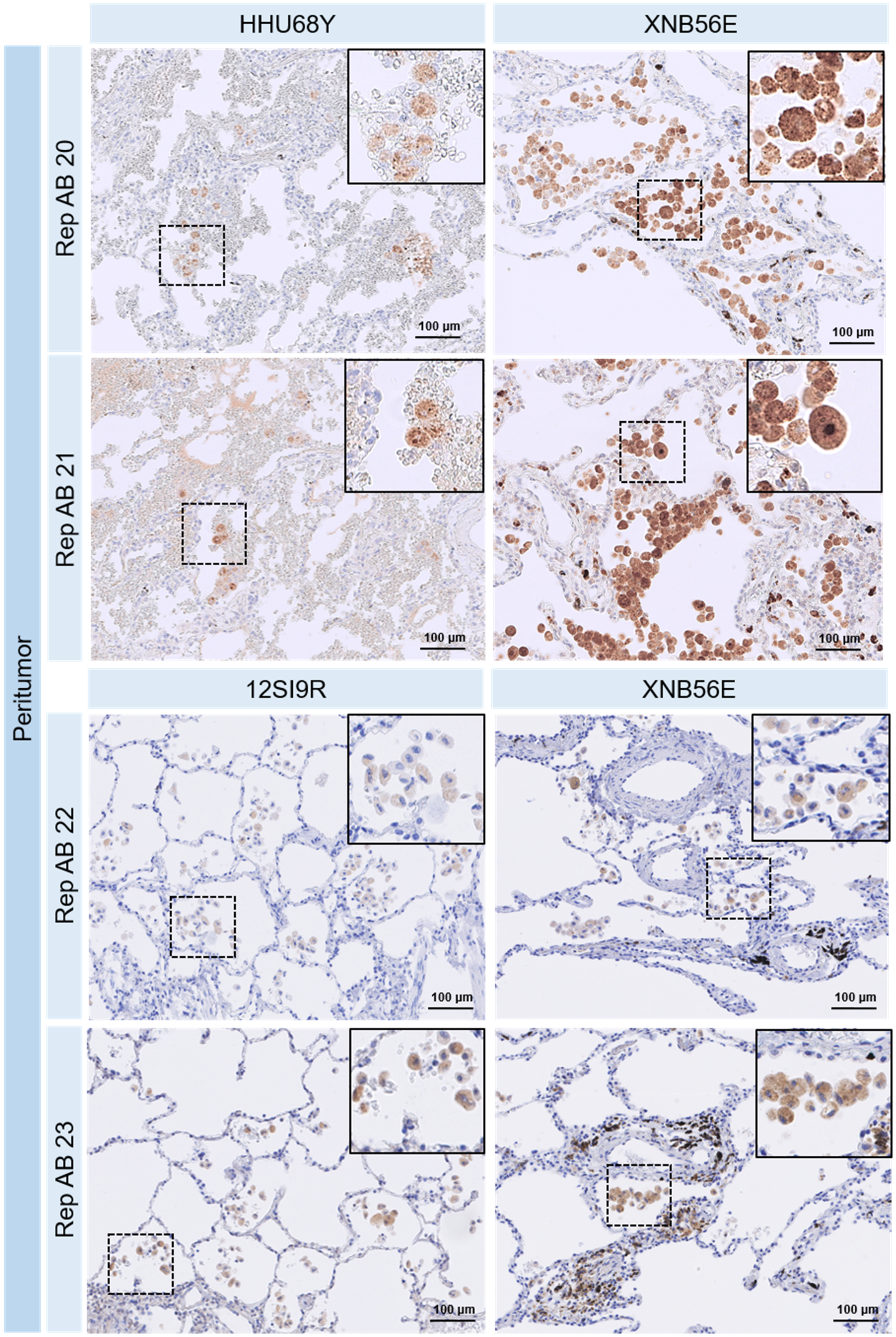

Figure S5. Immunohistochemical detection of BMMF Rep in peritumor tissues. DAB detection of lung peritumor tissue sections (FFPE) of 2 representative patients with Rep AB 20 and 21 (raised against C1HB.4 Rep) and AB 22 and 23 (raised against H1MSB.2, C1MI.3M.1, C1MI.9M.1 Rep).

Figure S6

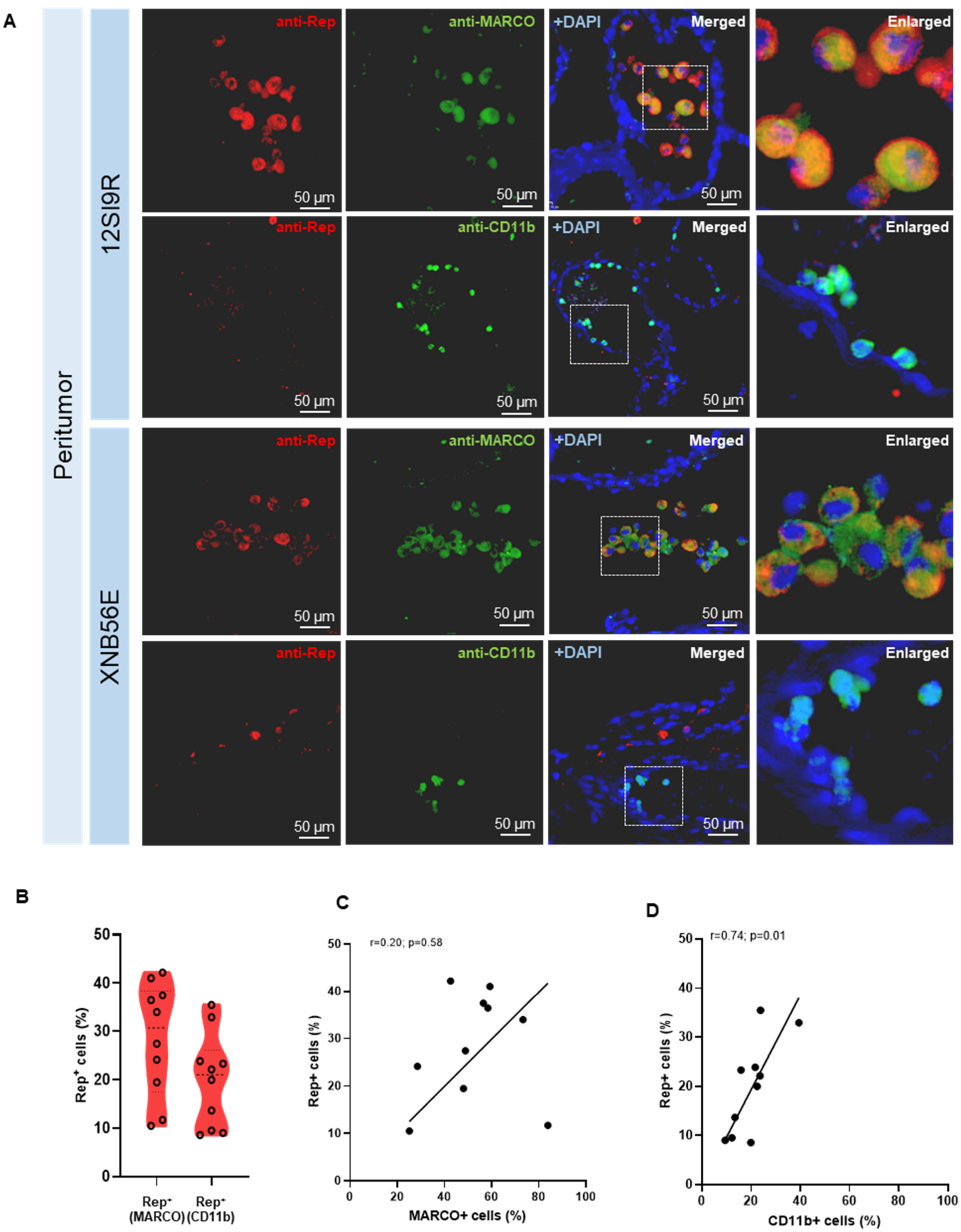

Figure S6. Co-immunofluorescence detection of Rep, MARCO and CD11b in peritumoral lung tissues. ***(A)*** Tissue sections from two representative lung cancer patients (peritumoral regions) stained by immunofluorescence microscopy using anti-Rep (AB3, red) in combination with either anti-MARCO or anti-CD11b antibodies (green), together with DAPI nuclear staining (blue). Merged images and enlarged views of selected regions (dashed boxes, two-fold enlargement) are shown. Scale bars, 50 µm. ***(B)*** Quantification of Rep^+ cells in the two individual tissue

staining setups with MARCO or CD11b in consecutive peritumoral tissues (n = 10 representative tissues). The slight observable difference between Rep positive cell numbers between MARCO and CD11b quantification setups may be explained by the preference for interstitial tissue parts for the CD11b quantifications in the individual cuts. Correlation plots for $MARCO^+$ ***(C)*** and $CD11b^+$ ***(D)*** cells in peritumor tissues plotted against the number of Rep^+ cells in individual patients (n=10).

Figure S7

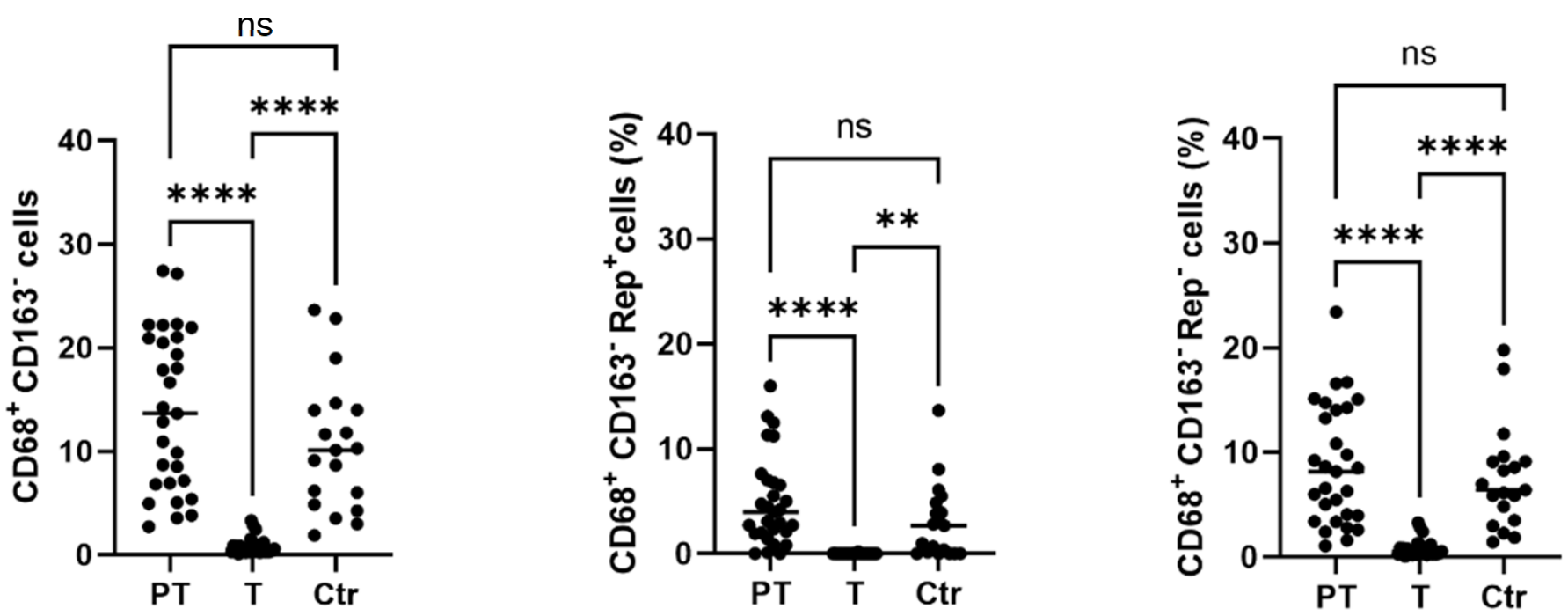

Figure S7. Quantification CD68^{+} CD163^{-} (M1-like) cells based on co-immunofluorescence microscopy. Semi-automated quantification of macrophages differentiated into CD68^{+}CD163^{-} M1-like cells (left) and further subgrouping into Rep^{+} M1-like macrophages (center) and Rep^{-} M1-like macrophages (right). One-way ANOVA test used for statistical analysis. Significance levels: *P<0.05, **P< 0.01, ***P<0.001, ****P<0.0001.

Figure S8

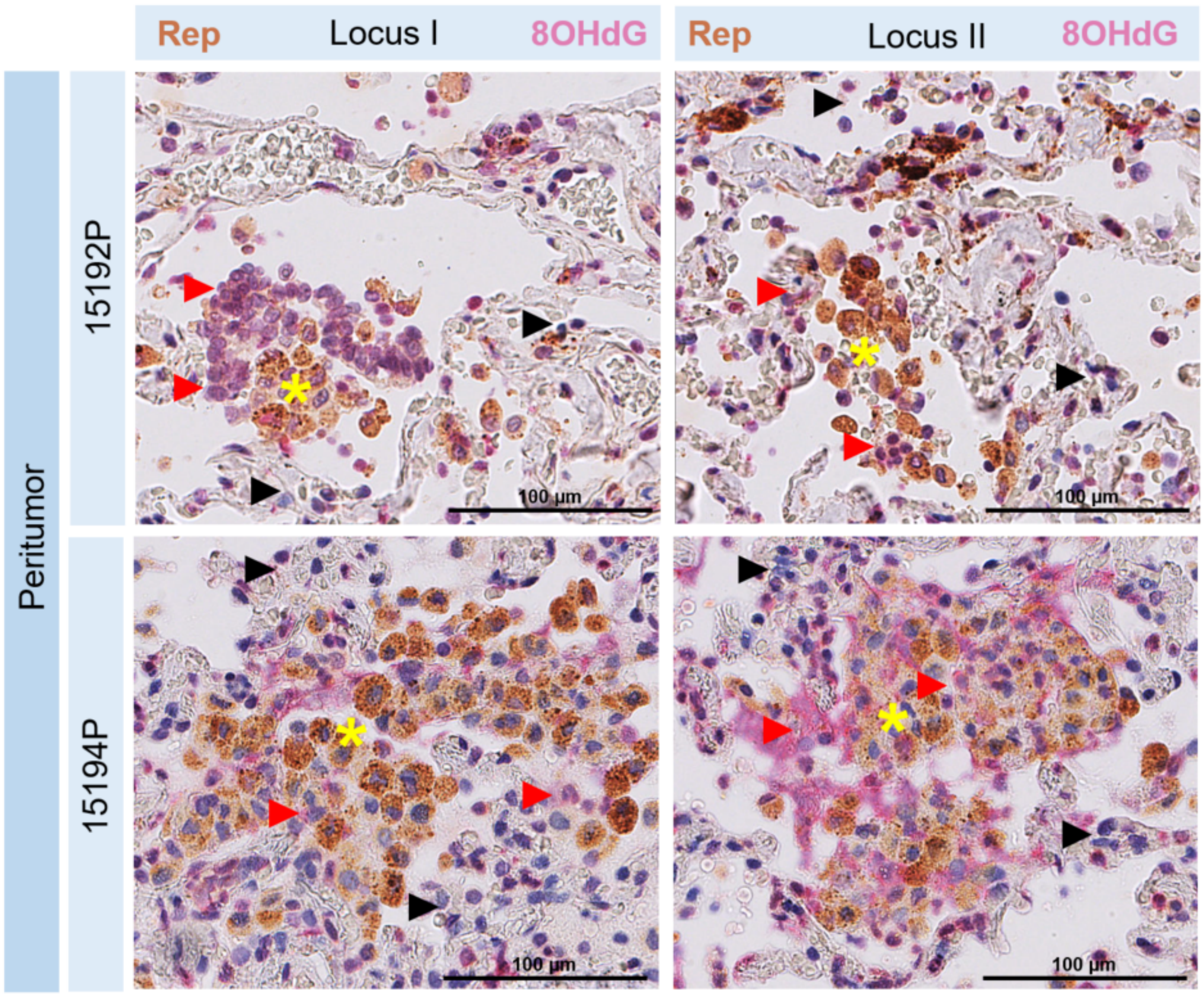

Figure S8. Immunohistochemical detection of BMMF Rep together with a marker of oxidative DNA damage. DAB anti-Rep (brown) co-detection together with a (surrogate) marker of oxidative DNA damage (8-Hydroxyguanosine, 8OHdG, red) in two representative peritumor tissues of lung cancer patients (two loci, each). Areas with increased densities of Rep- and 8OHdG-stained alveolar macrophages are marked with yellow asterisks, surrounding Rep-negative but 8OHdG-stained epithelial cells with red arrowheads and $Rep^{-}/8OHdG^{-}$ cells in the periphery with black arrowheads.

Figure S9

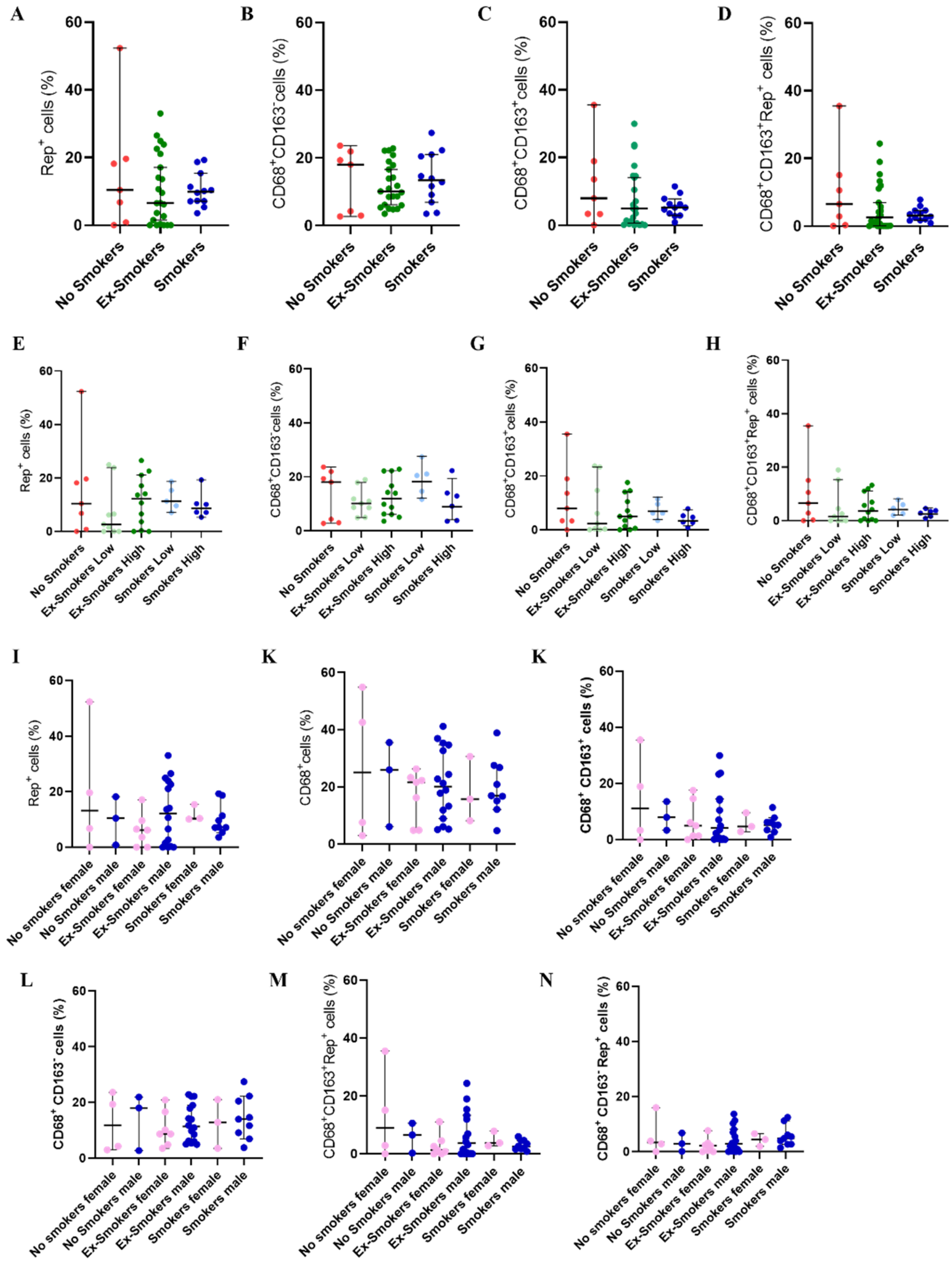

Figure S9. Rep expression in no smokers, ex-smokers and smokers including differentiation by sex. Scatter plots of Rep$^+$ cells **(A)**, CD68$^+$CD163$^-$ M1-like cells **(B)**, CD68$^+$CD163$^+$ M2-like cells **(C)** and Rep$^+$CD68$^+$CD163$^+$ cells **(D)** in no smokers (n=7), ex-smokers (n=23) and smokers (n=12). **(E-H)** Ex-smokers and smokers were divided into low and high-dose based on PY (pack-years – a clinical measure of tobacco exposure, >30 PY used as cut-off for high smokers/ex-smokers). **(I-N)** Scatter plots depicting Rep$^+$ cells **(I)**, CD68$^+$ cells **(J)**, CD68$^+$CD163$^+$ M2-like cells **(K)**, CD68$^+$CD163$^-$ M1-like cells **(L)**, Rep$^+$CD68$^+$CD163$^+$ cells **(M)** and Rep$^+$CD68$^+$CD163$^-$ cells **(N)** in no smokers, ex-smokers and smokers with further differentiation by sex. One-way ANOVA used for the statistical analysis. No significant differences detected.

Figure S10

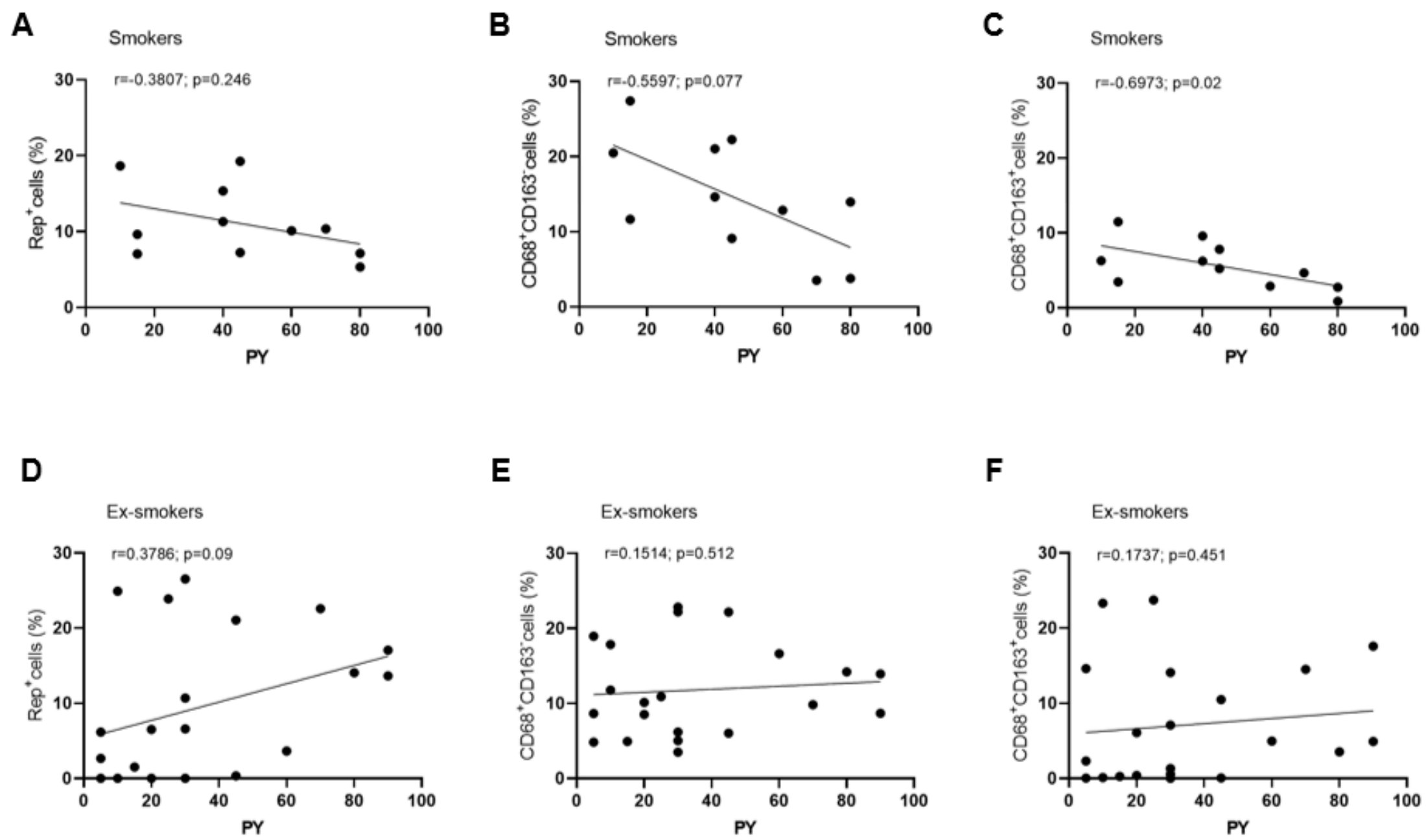

Figure S10. Correlation plots for the number of Rep$^+$ ***(A)***, CD68$^+$CD163$^-$ ***(B)*** and CD68$^+$CD163$^+$ ***(C)*** cells in peritumor tissues of actual smokers (n=11) and of Rep$^+$ ***(D)***, CD68$^+$CD163$^-$ ***(E)*** and CD68$^+$CD163$^+$ ***(F)*** cells in peritumor tissues of ex-smokers (n=21) plotted against the intensity of smoking (pack-years, PY) of the respective individual (statistical test by simple linear regression and Spearman's correlation).

Figure S11

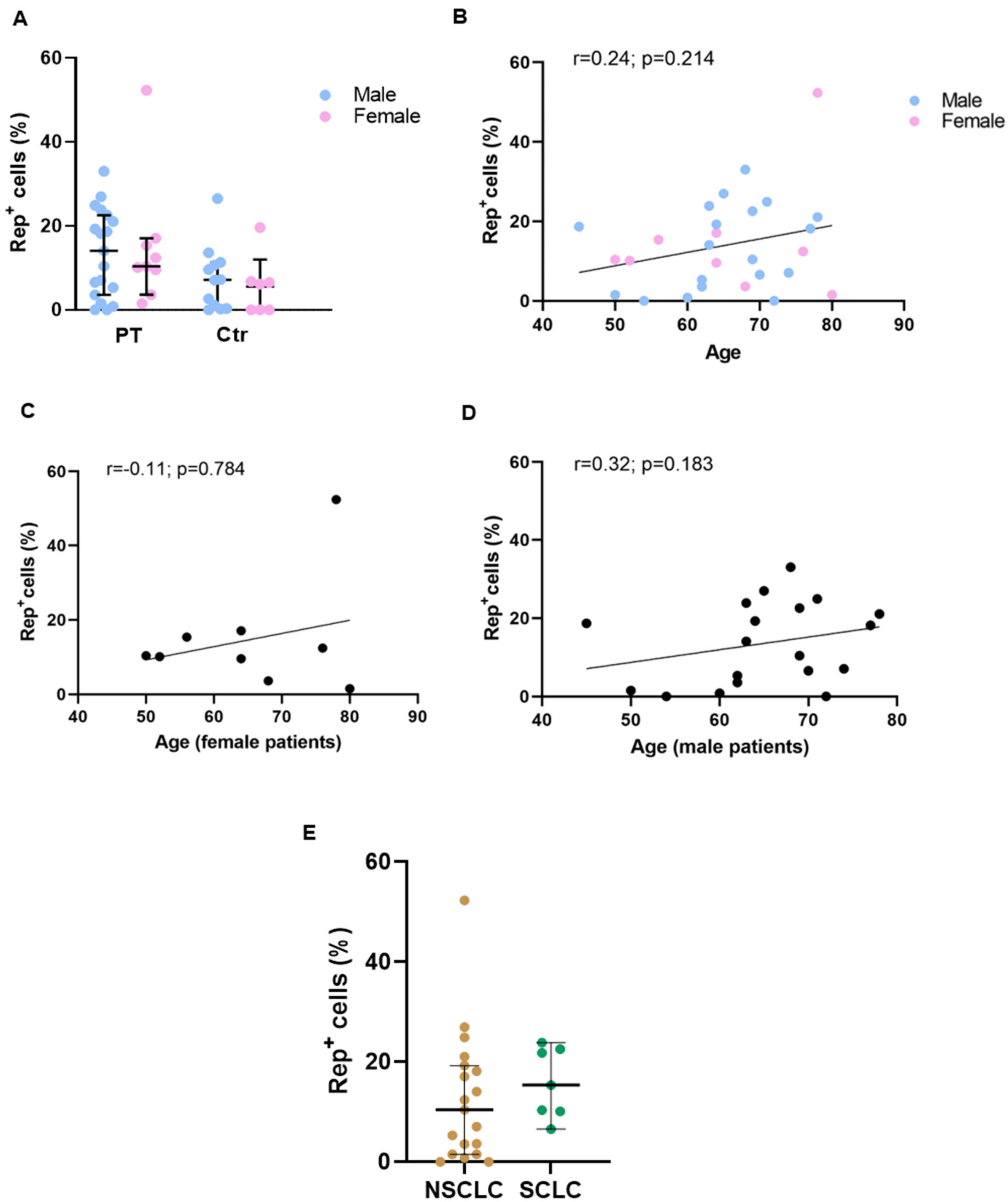

Figure S11. Rep correlation with clinical-epidemiological parameters. ***(A)*** Quantification of Rep^+ cells in peritumor (9 females, 19 males) and control tissues (7 females, 12 males) grouped by sex (Mann-Whitney test: ns). Correlations of Rep^+ cell numbers with age of female (n=9, pink) and male (n=19, blue) patients, together ***(B)***, or split for female ***(C)*** and male ***(D)*** patients (simple linear regression and Spearman correlation). ***(E)*** Stratification of Rep^+ cell for NSCLC (n=19) or SCLC (n=7) patients.

Figure S12

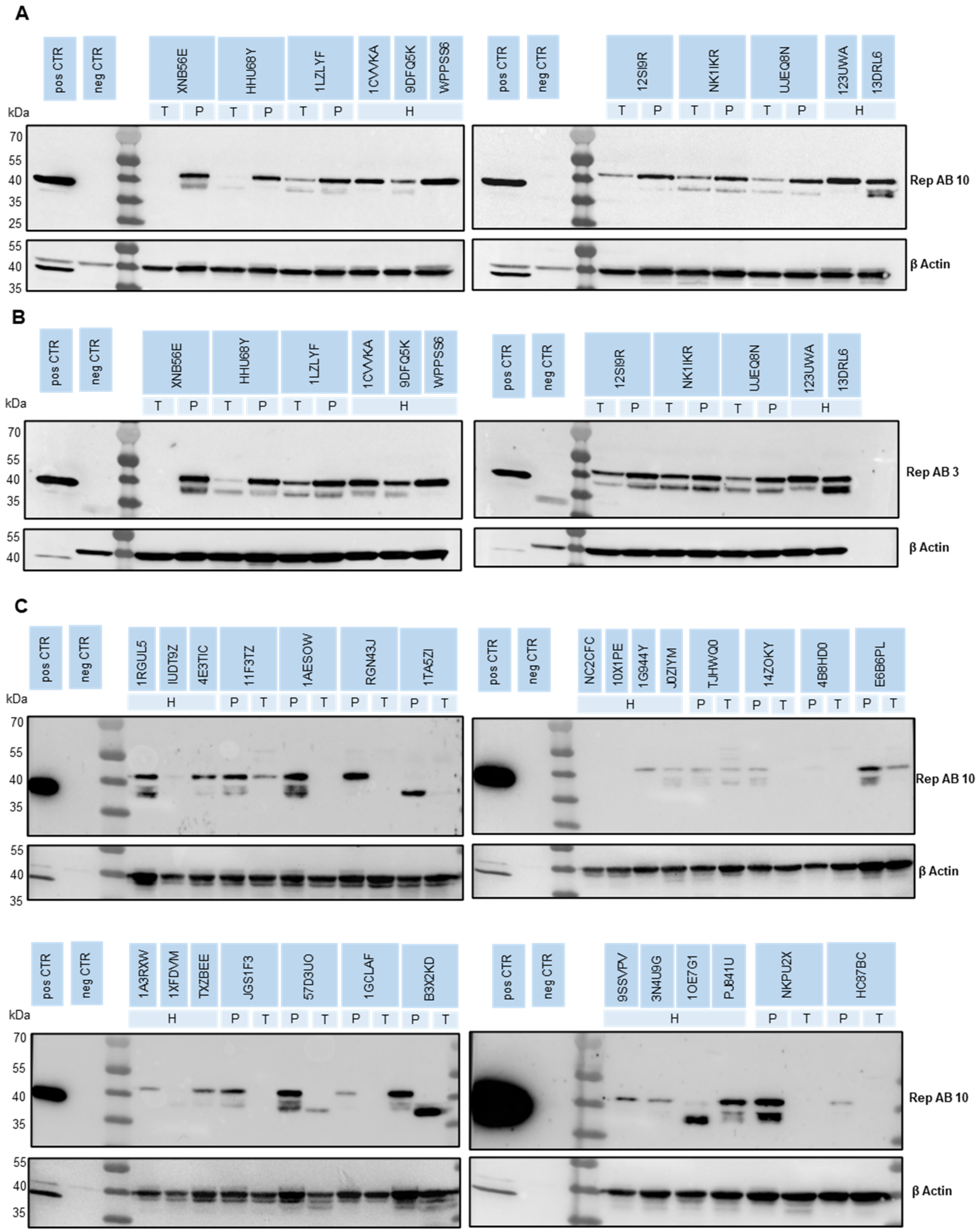

Figure S12. Immunoblotting based on lysates from paired lung cancer tumor and peritumor tissues and tissues adjacent to Hamartoma. ***(A)*** Immunoblotting of frozen lung samples from Thorax Klinik project 3359 with Rep AB 10 and β-actin. ***(B)*** Immunoblot of frozen lung samples from Thorax Klinik project 3359 with Rep AB 3 and β-actin (left: same blots as in Fig. 4, R, for comparison). ***(C)*** Immunoblot of frozen lung samples from Thorax Klinik project 3694 with Rep AB 10 and β-actin. 20 cancer patients and 19 non-cancer individuals tested, in total.

Figure S13

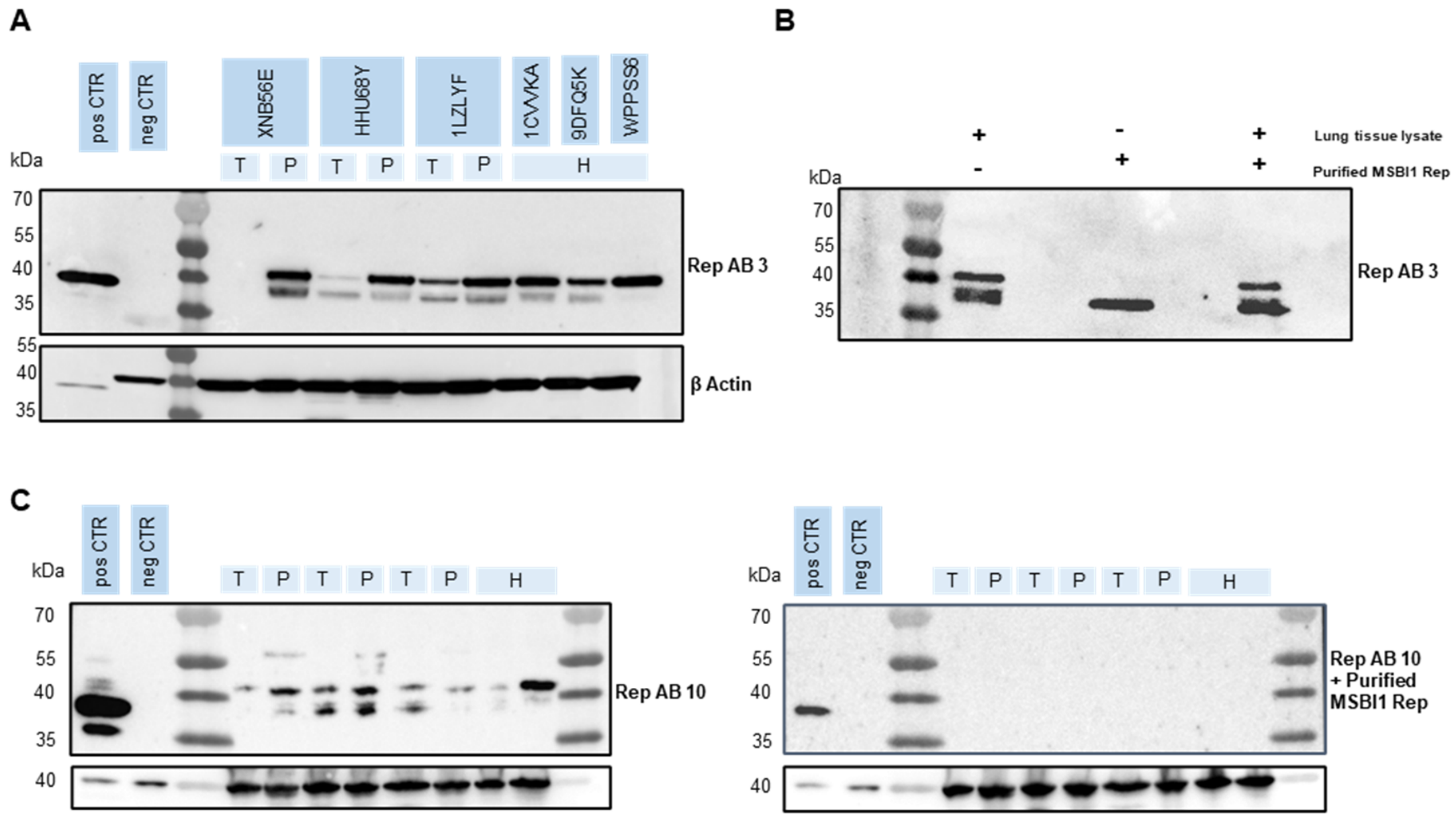

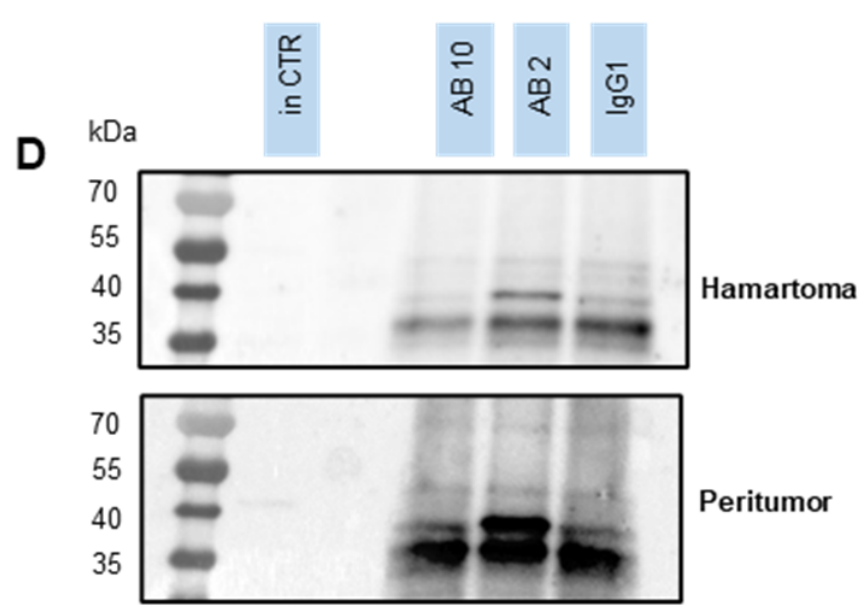

E

Sequence (23 aa)	BMMF1	Rep peptide?	Hits
LEEQFTQYDIEQISGLSSAYAVR	C1MI.2<br>C1MI.3M.1<br>C1MI.4<br>C1MI.5.1<br>C1MI.5.2N<br>H1MSB.1<br>Sphinx1.76<br>25BAMI	Yes	9 detection events

Figure S13. Detection of BMMF proteins by immunoblotting and mass spectrometry analysis. ***(A)*** WB results for Rep AB 3 for peritumor 'P' and tumor 'T' tissues from 3 lung cancer patients and 3 control tissues from non-cancer individuals (adjacent to Hamartoma, 'H', same data as in Fig. 4, R, for comparison). Frozen tissues used for lysate preparation. ***(B)*** Purified H1MSB.1 Rep protein was spiked into lung tissue lysates before immunodetection. The band of positive control overlaps the lower band at ~37 kDa observed for the tissue lysates. Rep AB 3 used for detection. ***(C)*** Blocking of anti-Rep antibody detection (4 µg/ml antibody AB 10) by co-incubation of WB membrane with purified H1MSB.1 Rep protein (17 ug/ml). ***(D)*** Detection of WB bands in one hamartoma and one peritumor tissue lysate after immunoprecipitation with Rep AB 10 or AB 2. ***(E)*** Mass spectroscopy-detectable BMMF1 Rep peptide (LEEQFTQYDIEQISGLSSAYAVR) identified in the peritumor sample also after immunoprecipitation with anti-Rep antibodies AB 2 and AB 10 (9 detection events).

Figure S14

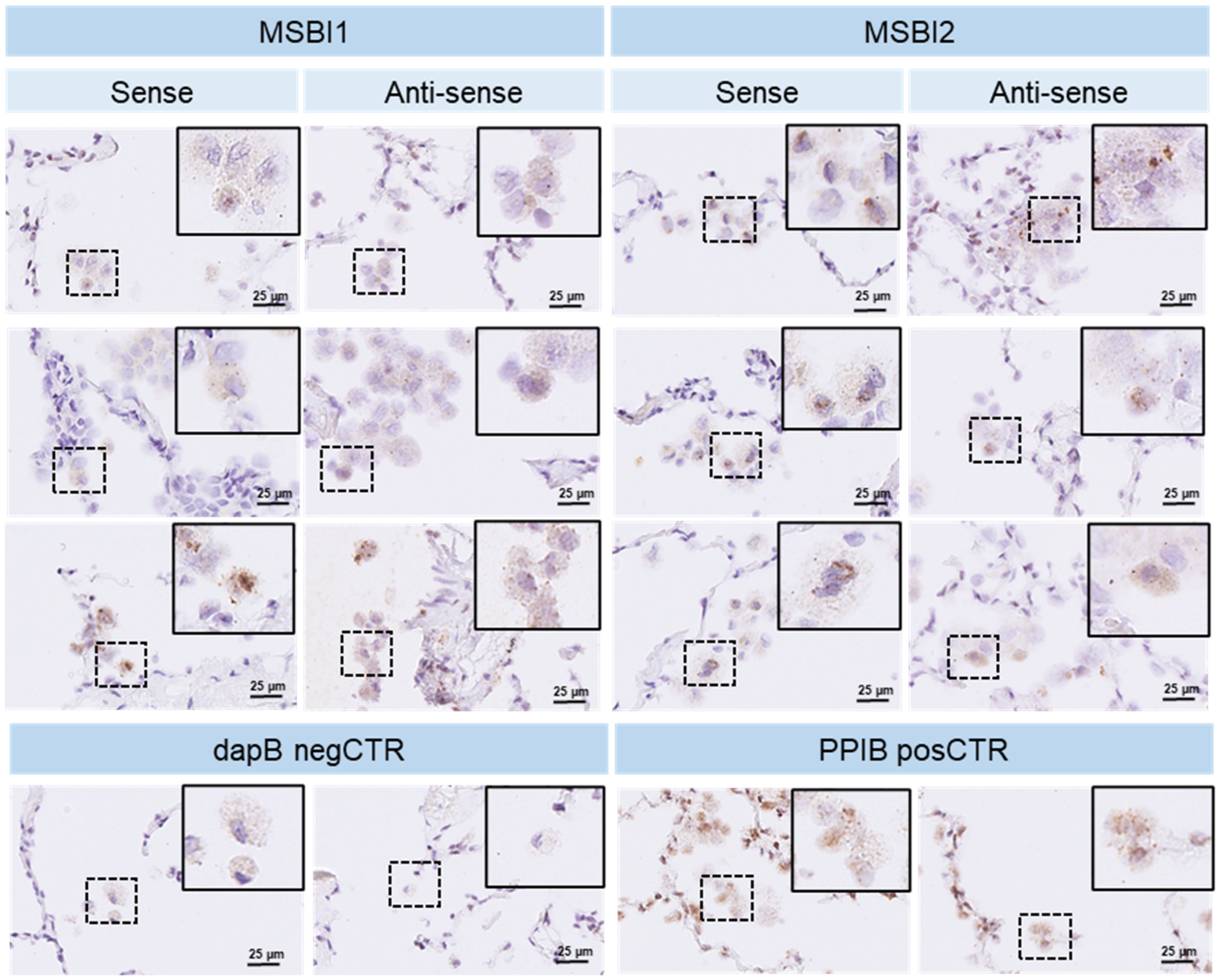

Figure S14. Histologic detection of BMMF RNA by *in situ* hybridization in peritumor tissues of lung cancer patients. Peritumor lung tissues (FFPE) stained with RNA sense and anti-sense probes directed against H1MSB.1 and H1MSB.2. Probes against dapB (4-hydroxy-tetrahydrodipicolinate reductase, negative) and PPIB (Peptidyl-prolyl cis-trans isomerase B, positive) were used as controls.

Figure S15

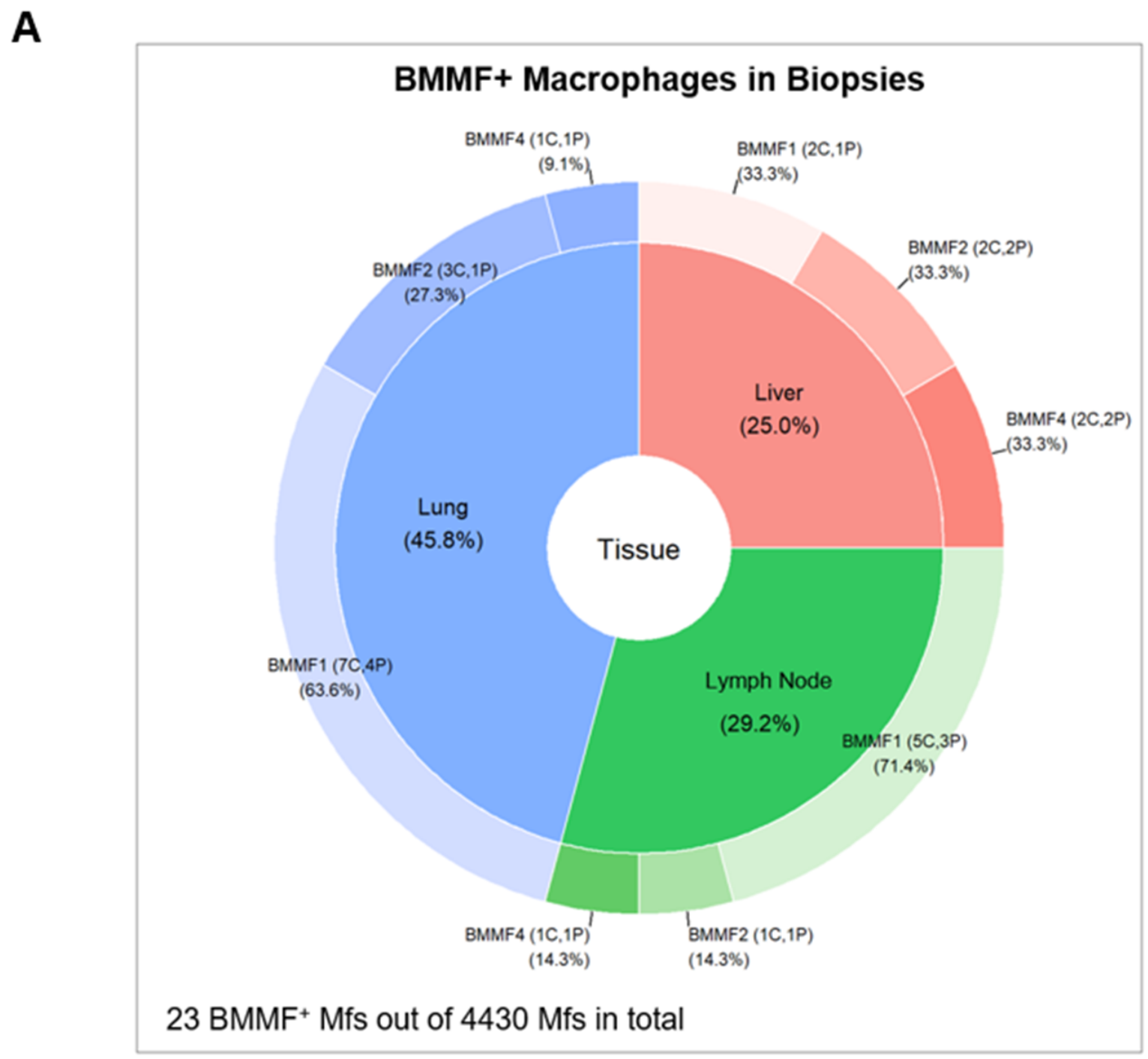

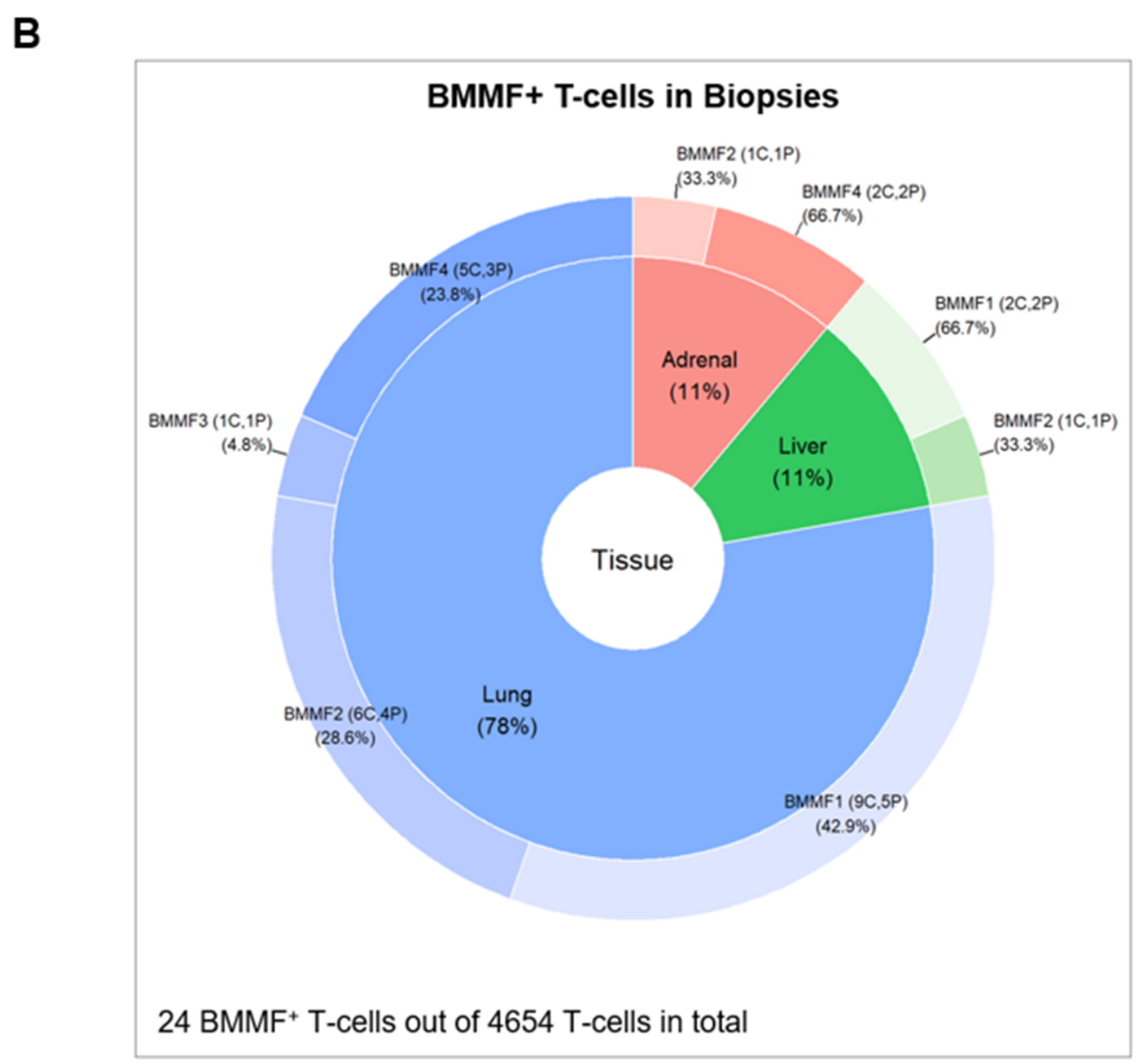

Figure S15. Characteristics of BMMF reads identified in macrophages and T-cells of lung cancer patients by scRNA-seq. Donut plots illustrating numbers of patients and cells harboring BMMF reads grouped into macrophages ***(A)*** and T-cells ***(B)*** prepared from biopsies of primary lung tumor and metastasis (C = cells, P = patients).
